# Enhancing encrypted HTTPS traffic classification based on stacked deep ensembles models

**DOI:** 10.1038/s41598-025-21261-6

**Published:** 2025-10-09

**Authors:** Ahmed M. Elshewey, Ahmed M. Osman

**Affiliations:** 1 Department of Computer Science, Faculty of Computers and Information, Suez University, P.O.Box:43221, Suez, Egypt; 2 Department of Information Systems, Faculty of Computers and Information, Suez University, P.O.Box:43221, Suez, Egypt

**Keywords:** HTTPS traffic classification, Encrypted traffic, Network security, Network traffic classification, Deep learning, Ensemble learning, Cyber security, CNN, DNN, Engineering, Mathematics and computing

## Abstract

The classification of encrypted HTTPS traffic is a critical task for network management and security, where traditional port or payload-based methods are ineffective due to encryption and evolving traffic patterns. This study addresses the challenge using the public Kaggle dataset (145,671 flows, 88 features, six traffic categories: Download, Live Video, Music, Player, Upload, Website). An automated preprocessing pipeline is developed to detect the label column, normalize classes, perform a stratified 70/15/15 split into training, validation, and testing sets, and apply imbalance-aware weighting. Multiple deep learning architectures are benchmarked, including DNN, CNN, RNN, LSTM, and GRU, capturing different spatial and temporal patterns of traffic features. Experimental results show that CNN achieved the strongest single-model performance (Accuracy 0.9934, F1_macro 0.9912, ROC-AUC_macro 0.9999). To further improve robustness, a stacked ensemble meta-learner based on multinomial logistic regression was trained on model outputs, achieving state-of-the-art performance with Accuracy 0.9949, Precision_macro 0.9923, Recall_macro 0.9941, F1_macro 0.9932, and ROC-AUC_macro 0.9998. The framework also outputs confusion matrices, ROC curves, and learning curves for interpretability. To ensure reproducibility and practical use, the full codebase is publicly available on GitHub, providing researchers and practitioners with a deployment-ready pipeline for encrypted traffic analytics where ensemble learning surpasses individual models.

**Supplementary Information:**

The online version contains supplementary material available at 10.1038/s41598-025-21261-6.

## Introduction

Modern traffic classification has outgrown traditional port or payload inspection techniques because widespread encryption renders packet contents opaque and application ports dynamic. Recent surveys emphasize that, despite encryption, flow-level features and machine-learning pipelines can still characterize and classify traffic accurately. These works position machine learning (ML) as the practical path for encrypted traffic analysis, outlining end-to-end procedures and state-of-the-art methods that operate without decrypting content^1–3^.

In parallel, research in software-defined networking (SDN) highlights how separating control and data planes creates a programmable substrate where ML-based classifiers can improve quality of service, automate management, and strengthen security. SDN-integrated ML report consistent outperformance over traditional traffic-classification methods and identify scalability and large-scale deployment as ongoing challenges^2,4^. These trends motivate experiments on public HTTPS traffic corpora with multi-class labels and class imbalance, aligning model choices (CNN/LSTM/RNN/DNN) and ensemble stacking with the literature’s guidance on encrypted-traffic ML and SDN-aware classification^5^.

Encrypted HTTPS traffic is increasingly difficult to classify because payloads are opaque, ports are unreliable indicators of application intent, and real-world datasets exhibit severe class imbalance^6^. The practical problem is to design a reproducible, deployment-ready pipeline that, using only flow-level features, achieves high macro performance across six HTTPS categories (D, L, M, P, U, W) despite imbalance and inter-class similarity, while remaining robust to distribution shifts. Existing single-model approaches often peak on the majority class and underperform in minority classes; therefore, a principled comparison of deep architectures (DNN, CNN, RNN, LSTM and GRU) and an ensemble strategy that fuses their complementary strengths is needed. The task is to build such a pipeline on a public Kaggle dataset, automate label normalization and stratified splitting, mitigate imbalance during training, and deliver a validated model that improves macro-F1 and accuracy beyond individual baselines.

The main contribution of this work is the development of a complete, reproducible pipeline for encrypted HTTPS traffic classification using a public Kaggle dataset (145,671 flows, 88 features) that contains six canonical application categories. Unlike prior efforts that rely on manual feature engineering or focus on a single architecture, this study introduces (1) an automated preprocessing stage that detects the label column, maps heterogeneous label values to standardized categories, performs stratified data splitting, and applies feature scaling; (2) a comprehensive benchmarking of deep models (DNN, CNN, RNN, LSTM, and GRU) under imbalance-aware training; and (3) a stacked ensemble meta-learner that integrates the probabilistic outputs of the base models, achieving state-of-the-art performance with Accuracy 0.9949 and Macro-F1 0.9932. To ensure transparency and reproducibility, the full codebase is released on GitHub, providing the community with a ready-to-use framework for HTTPS traffic analytics and a baseline for future research on encrypted traffic classification.

Our contribution is a deployment-oriented, leakage-controlled recipe for flow-level encrypted HTTPS classification, standardizing the entire pipeline, implementing probability-level stacked ensembling, conducting a like-for-like comparison of deep architectures under the same dataset, splits, preprocessing, imbalance handling, and training budget, achieving state-of-the-art results on the same HTTPS/CESNET family, and emphasizing reproducibility and deployment.

The remainder of this paper is organized as follows. Section 2 reviews related work on encrypted traffic classification and the role of machine and deep learning in modern network analytics. Section 3 describes the dataset obtained from Kaggle, outlines its characteristics, and explains the preprocessing pipeline including label normalization, stratified splitting, scaling, and imbalance handling. Section 4 reports the experimental results, comparing the performance of individual models with the stacked ensemble, and visualizing outcomes using confusion matrices, ROC curves, and learning curves. Finally, Sect. 5 concludes the paper and outlines future research directions.

## Related works

Encrypted traffic classification has become a central focus in network security and management due to the widespread adoption of protocols such as HTTPS, TLS, and VPN, which obscure packet payloads and render traditional port or signature-based methods ineffective. This section reviews some studies related to traffic classification.

Wang et al.^7^ have developed a federated learning framework for traffic classification in smart home networks, addressing privacy, labeling cost, and data dependency issues. The framework includes a DPI-based labeling mechanism at edge gateways, a semi-supervised model built on autoencoders, and explainable AI techniques. Evaluations show strong classification accuracy with few samples while maintaining privacy.

Wang and Gu^8^ addressed the limitations of traditional and even many deep learning methods in encrypted traffic classification, particularly regarding high computational resource demands and limited interpretability. They proposed a Parameter-Efficient Fine-Tuning (PEFT) strategy to reduce the number of parameters that need adjustment during training while maintaining competitive performance. The approach was validated across multiple public datasets and tasks, including Tor service classification and malicious traffic detection, and was benchmarked against state-of-the-art deep models. Results demonstrated that the method significantly lowered memory and computation costs without sacrificing accuracy. In addition, their analysis of the pre-trained model revealed a hierarchical and structured representation of encrypted traffic, offering valuable interpretability.

Liu et al.^9^ proposed a hybrid deep learning architecture (ATVITSC) designed to overcome the limitations of traditional and feature-engineered approaches for encrypted traffic classification. Their method introduces a two-stage framework: in preprocessing, payloads are transformed into packet-level images and aggregated into session-level representations; in classification, features are jointly captured through two complementary modules. The Packet Vision Transformer (PVT) leverages multi-head self-attention to extract global dependencies, while the Spatiotemporal Feature Extraction (STFE) module combines convolution with attention for spatial patterns and a Bi-LSTM for temporal dynamics. A feature fusion module then adaptively weights and integrates global and spatiotemporal features for final classification. Experiments across multiple encrypted traffic domains show that ATVITSC achieves strong macro-F1 scores (ranging from 94.9% to 99.7%), consistently outperforming state-of-the-art baselines and demonstrating superior generalization.

Park et al.^10^ advanced encrypted traffic classification by introducing a multi-task learning framework that jointly trains multiple related classification objectives within a single model. Their approach was validated on the ISCX 2016 VPN/Non-VPN dataset, which contains three classification tasks: encapsulation, category, and application identification. By sharing representations across tasks, the model achieved high efficiency and strong performance, with reported accuracies of 99.29%, 97.38%, and 96.89% on the respective tasks. Compared with conventional single-task and non-lightweight methods, the proposed multi-task strategy not only improved predictive accuracy but also enhanced computational efficiency, demonstrating the value of jointly optimizing correlated classification problems in encrypted traffic analysis.

Mahboob and Chung^11^ examined the growing complexity of encrypted traffic classification in the context of network monitoring, QoS management, and intrusion detection, where conventional approaches often fail to adapt to evolving protocols and encrypted flows. They proposed a deep learning–based method for categorization and application service classification, evaluating both DNN and CNN models under different feature selection strategies and TCP flow timeouts. Their experiments in a software-defined wireless network (SDWN) setting demonstrated that a DNN with information gain features and a 60-second timeout achieved near-perfect performance, with 100% training accuracy, 99.9% testing accuracy, and minimal generalization gap (0.001). In contrast, CNN performed less effectively, with accuracy ranging from 84% to 90% depending on timeout and features. Importantly, their DL approach significantly outperformed existing baselines, with improvements of up to 86% in F1-score, 64% in precision, and 21% in recall, highlighting the strength of feature selection and timeout optimization in enhancing encrypted traffic analysis.

Liu et al.^12^ addressed the data scarcity and labeling burden inherent in deep learning–based traffic classification. Since creating large, annotated datasets is costly and time-intensive, they proposed MTEFU, a multi-task learning framework designed to reduce dependence on extensive labeled data. The method integrates multiple related tasks including flow duration, bandwidth size, and business traffic category into a shared-parameter deep learning model, enabling information transfer across tasks. Several neural architectures, including CNN, SAE, GRU, and LSTM, were employed within this framework and evaluated on the QUIC dataset. Results showed that MTEFU achieved 94.67% accuracy with only 150 labeled samples, performance comparable to single task learning models trained with a fully labeled dataset of over 6,000 samples.

Belkadi et al.^13^ explored traffic classification within SDN and cloud environments, emphasizing its importance for network security, anomaly detection, and QoS management. Their study demonstrated that incorporating traffic classification into SDN can optimize flow management by aligning with application requirements. They evaluated multiple supervised machine learning algorithms (Naive Bayes, SVM (SMO), Random Forest, and C4.5 (J48)) using both raw features and features generated during experimentation. The results showed promising performance, achieving up to 97% accuracy with the studied features and over 95% accuracy with generated features.

Pathmaperuma et al.^14^ explored privacy and security risks in mobile applications by proposing a deep neural network (DNN)-based framework to detect fine-grained in-app user activities from encrypted Internet traffic streams. Since it is infeasible to collect training data from all possible applications, the framework leverages the probability distribution of the DNN output layer to identify and filter unknown traffic corresponding to applications not seen during training. A time windowing approach is also employed to segment traffic flows, enabling activity recognition from only a fraction of the session’s encrypted packets. Experiments demonstrated that the framework achieved over 90% accuracy in recognizing in-app activities for applications included in training, while maintaining an average accuracy of 79% in detecting unseen activities as unknown.

Ismaeel et al.^15^ investigated traffic pattern classification in smart cities, proposing a deep recurrent neural network (RNN) based approach capable of capturing the dynamic and sequential properties of traffic flows. Their model integrates convolutional layers for feature extraction with recurrent layers to learn temporal dependencies, followed by a SoftMax layer for classification. Evaluated on a real-world smart city traffic dataset, the method outperformed traditional and baseline approaches across accuracy, precision, recall, and F1 score. Reported precision reached up to 95%, demonstrating strong predictive ability and robustness. Beyond performance gains, the study also analyzed results in depth and discussed implications for traffic management in urban environments, emphasizing that deep recurrent architectures can significantly improve the accuracy of smart city traffic monitoring systems.

Sun et al.^16^ addressed the growing challenge of traffic classification under VPN and TLS encryption, which conceals payload data and complicates analysis. Leveraging advances in deep learning for image recognition, they introduced a method based on the novel concept of a Packet Block, defined as an aggregation of continuous packets in the same direction. These packet blocks are converted into images, from which features are extracted and then classified using convolutional neural networks (CNNs). Experiments were conducted on both a captured OpenVPN dataset and the public ISCX-Tor dataset, achieving classification accuracies of 97.20% and 93.31%, respectively. The results outperformed state-of-the-art methods, demonstrating that packet block image representation combined with CNNs is an effective approach for encrypted traffic classification in VPN and TLS contexts.

Shi et al.^17^ recognized the shortcomings of existing encrypted traffic classification methods, where traditional ML depends heavily on feature engineering, deep learning approaches often require large amounts of labeled data, and pretrained models overlook local traffic features. To address these gaps, they proposed the BERT-based Byte-level Feature Convolutional Network (BFCN), which integrates global and local traffic representations. The model consists of two modules: a BERT-based packet encoder that captures global features through attention mechanisms, and a CNN module that extracts byte-level local features. These representations are concatenated to form a richer traffic embedding. Evaluated on the ISCX-VPN dataset, BFCN achieved state-of-the-art F1 scores of 99.11% (service classification) and 99.41% (application classification), outperforming previous benchmarks.

Lu et al.^18^ tackled the challenge of encrypted traffic classification by proposing the Inception-LSTM (ICLSTM) model, which integrates Inception modules with LSTM layers to capture both hierarchical and sequential features of traffic flows. Their method converts raw traffic data into grayscale images, allowing the model to extract discriminative spatial and temporal features simultaneously. To address class imbalance, they introduced class-specific weighting during training, ensuring balanced recognition across minority and majority traffic types. Validated on the ISCX 2016 dataset, ICLSTM achieved over 98% accuracy for both standard encrypted traffic and VPN-encrypted traffic service identification. This framework not only improves accuracy but also simplifies feature engineering, demonstrating that deep learning can both enhance performance and reduce manual overhead in encrypted traffic analysis.

Chen and Wang^19^ address the limits of single-feature encrypted-traffic classifiers by proposing MPAF (Multi-Phase Attribute Fingerprint). MPAF stages inference in three phases aligned with different periods of a TLS session and embeds discrete attributes into vectors, then fuses decisions via a leaf-node masking tree tailored to the multi-phase setup. Experiments report 96.33%–99.42% accuracy with average waiting time 0.18–0.45 s, indicating a favorable accuracy–latency balance. The system is especially robust under practical stressors, outperforming prior methods with small training sets, in cross-dataset tests, and for unknown application recognition.

Liu et al.^9^ tackled the difficulty of classifying today’s encrypted traffic by introducing ATVITSC, a hybrid vision/sequence framework. They first convert each session into a single session image by combining packet-level images derived from payloads, reducing cross-packet ambiguity. Classification then runs in two parallel streams: a Packet Vision Transformer (PVT) with multi-head self-attention to capture global, long-range structure, and a Spatiotemporal Feature Extraction (STFE) path that uses convolution with attention for spatial cues and a bidirectional LSTM to model temporal relations across packets. A feature-fusion classifier applies dynamic weighting to merge both representations for the final decision. Across diverse encrypted settings (VPN, Tor, malicious, and mobile traffic) ATVITSC attains macro-F1 of 97.88%, 98.79%, 99.67%, and 94.90%, respectively, outperforming contemporary methods and demonstrating stronger generalization.

Malekghaini et al.^20^ examine the real-world durability of deep encrypted-traffic classifiers by tracking two state-of-the-art models across multiple ISP datasets collected over two years, where protocol evolution and application updates induce data drift that erodes accuracy. Rather than relying on lab-style corpora, they quantify how service-level and application-level performance changes as patterns shift, showing why results reported on controlled datasets can be over-optimistic for production. The paper then proposes practical adaptation guidelines and argues these recommendations generalize across encryption protocols and label granularities. The core contribution is an evidence-based blueprint for keeping deployed ETC models reliable as traffic and protocols evolve.

Wang et al.^21^ argue that single-modality encrypted-traffic classifiers miss crucial cross-flow context and propose MeDF, a multimodal model that fuses intra-flow and inter-flow information. Intra-flow cues are built by turning raw bytes into spectrograms and pairing them with statistical features, while inter-flow context is captured via a flow-relation graph that models dependencies among flows. A fusion module leverages these complementary views to extract more usable signal from encrypted traffic than either view alone. Evaluated on two real-world datasets, MeDF achieves 98.57% and 94.73% accuracy, outperforming both classical single-modality baselines and recent multimodal methods, and demonstrating that combining intra-flow representations with inter-flow relations overcomes key limitations of prior approaches.

Yan et al.^22^ target line-rate classification in high-speed networks, where most deep or feature-heavy methods are too slow, by proposing HETC, a two-stage pipeline optimized for short flows. Stage 1 quickly decides whether traffic is encrypted by sampling short flows and computing aggregation entropies plus chi-square-based byte-distribution features, separating encrypted from cleartext without waiting for full flows. Stage 2 adds lightweight binary payload features and performs fine-grained application classification with a Random Forest, keeping inference cost low. On a 1 KB flow-length setting, HETC reports 94% F-measure for encryption detection and 85–93% F-measure for fine-grained classes, while processing each flow in ~ 2–16 ms making it a practical candidate for high-speed encrypted traffic classification.

Chen et al.^23^ proposed a lightweight graph–based encoder for encrypted-traffic classification that tackles unclear local features and low accuracy in legacy detectors. They convert packet byte sequences into byte-level traffic graphs, apply a weight-matrix–based output to slim the model, and feed a pipeline with (i) an embedding layer, (ii) a GraphSAGE encoder (sampling/averaging) to produce per-packet representations, and (iii) a time-information extractor that separately embeds headers and payloads. For end-to-end learning, the graph features are fused with an enhanced Transformer using relative positional encoding to capture temporal order, yielding the final predictions. Evaluated on WWT, ISCX-2012, and ISCX-Tor, the method outperforms 12 + baselines, reaching F1 = 0.9938 (ISCX-2012) and 0.9856 (ISCX-Tor). Ablations show an 18.2% parameter reduction versus TFE-GNN while improving accuracy, indicating the encoder enhances application and anomaly detection effectiveness without incurring heavy model size.

Xu et al.^24^ target few-shot traffic classification, where standard two-layer GCNs underperform due to over-smoothing and the distortions introduced by zero-padding short flows to a fixed length. They propose ADGCN, an end-to-end method that first uses an autoencoder (AE) to reconstruct short flows by learning abstractions from longer flows of the same class, replacing zeros with informative representations and preserving within-class structure. The reconstructed sequences are then classified with GCNII, a deeper graph convolutional network designed to avoid over-smoothing and better capture higher-order relationships among flows. Across few-shot settings, ADGCN delivers 3.5%–24% accuracy gains over contemporary methods, showing that combining AE-based reconstruction with a deep (rather than shallow) GCN resolves key limitations of prior GCN-based traffic classifiers under limited data.

Additionally, recent 2025 studies push encrypted-traffic classification on several fronts: adversarially pre-trained transformers that improve cross-dataset robustness^25^ ; hierarchical/incremental frameworks that evolve with a flow and better handle drift^26^; explainable path-signature features that make decisions more interpretable^27^; dual-modal fusion models that combine statistical and learned representations for stronger accuracy^28^; lightweight cross-modal designs that target practical throughput and deployment cost^29^; systematic analyses of CNN complexity–performance trade-offs for QUIC traffic^30^; and multi-task DoH models that jointly learn detection and family classification under encryption^31^. Together, these works underscore the field’s shift toward robustness, interpretability, and efficiency. This paper distinguishes itself from prior studies by systematically benchmarking multiple deep learning models and introducing an ensemble framework specifically tailored for encrypted HTTPS traffic classification. Using the publicly available Kaggle HTTPS Traffic Classification dataset (145,671 flows, 88 features, and six classes: Download, Live Video, Music, Player, Upload, Website), the pipeline first automates preprocessing, including label detection, normalization, stratified splitting, and imbalance-aware weighting. Unlike studies that focus on single architecture or specialized tasks (e.g., DoH detection, Tor traffic, or activity inference), this work evaluates DNN, CNN, RNN, LSTM, and GRU models under a unified framework, highlighting both their strengths and weaknesses. The experiment then introduces a stacked ensemble meta-learner (multinomial logistic regression) that learns optimal weights from base model predictions, thereby mitigating class imbalance and maximizing macro-level performance. In this way, the experiment contributes a comprehensive, reproducible, and ensemble-driven methodology that unifies diverse deep learning paradigms, extending the scope of prior research to a broader and more practical encrypted traffic classification scenario.

Additionally, the study standardizes the tabular flow setting for HTTPS application categories and makes the stacked-probability protocol explicit and leakage-free. This addresses a gap in literature for reproducible, operations-ready pipelines for encrypted traffic without payloads, where consistent performance on minority classes and transparent comparisons matter more than architectural novelty. Table 1 summarizes some studies related to HTTPS traffic classification and relation with this work.

**Table 1:** Summary of some studies related to HTTPS traffic classification and relation with this work.

Study	Contribution	Input and learning style	Performance	Relation to our work
Wang et al.^7^	Federated learning for smart-home ETC; DPI-assisted labeling; semi-supervised AE; XAI	Edge-federated; flow/packet + DPI labels	Strong accuracy with few samples; privacy preserved	Complementary: our stack can be trained per-site and federated; our flow-only features avoid DPI; we add calibration + gated deployment.
Wang & Gu^8^	PEFT for encrypted traffic (parameter-efficient fine-tuning) with interpretability of pretrain	Pretrained backbone + PEFT; low compute	Matches state of art (SOTA) with far fewer trainable params	Orthogonal: PEFT can shrink our bases or become a base in the stack; we supply the leakage-controlled eval + deployment blueprint.
Liu et al. (ATVITSC)^9^	Packet-image ViT + STFE (Conv + Attn + BiLSTM) with adaptive fusion	Payload→images; hybrid vision/sequence	Macro-F1 ≈ 95–100% (domain-dependent)	Contrast: high accuracy, heavier payload imaging; our flow-only stack is lighter/privacy-friendlier; ATVITSC could be added as a base if payload is allowed.
Park et al.^10^	Multi-task learning (encapsulation, category, app) on ISCX2016	Shared rep; multi-head	Acc. 99.29/97.38/96.89%	Complementary: MTL could supply a shared encoder feeding our stack; we focus on calibrated single-task outputs and deployment knobs.

Encrypted traffic exposes multi-scale structure: short-range bursts in packet size and timing, medium-range ordering of segments, and global rate/ratio aggregates at the flow level. Single architectures emphasize different parts of this structure (e.g., CNNs capture local burst motifs; GRU/LSTM learn sequence order and duration; DNN/MLP models non-linear interactions among aggregates). Stacked ensembling exploits this complementarity by training diverse base learners and learning a meta-model on their out-of-fold predictions. In our setting, stacking reduces residual confusions among semantically adjacent classes (e.g., Website/Music/Player) where any one inductive bias is insufficient.

Bagging averages replicas of the same learner to reduce variance; boosting reweights hard examples to reduce bias but can be sensitive under class imbalance. Stacking learns how to combine heterogeneous learners, letting the meta-model emphasize whichever base is most reliable in each region of the feature space (e.g., favor CNN near bursty patterns, GRU near ordered segments, DNN when global ratios dominate). This is particularly effective on flow-level inputs where we avoid payload inspection and must leverage timing/size signals efficiently.

A common pitfall is training the meta-model on base predictions from the same examples those bases were fit on. We therefore use K-fold out-of-fold (OOF) stacking: each base predicts held-out folds; the meta-model trains only on OOF predictions and are evaluated on a disjoint test set. We choose multinomial logistic regression as the meta-learner because it is calibration-friendly, has microsecond-level overhead, and yields interpretable coefficients over base logits. Post-hoc temperature scaling on validation further improves calibration; per-class thresholds then tailor precision/recall to operational costs.

## Methodology

**Fig. 1:**
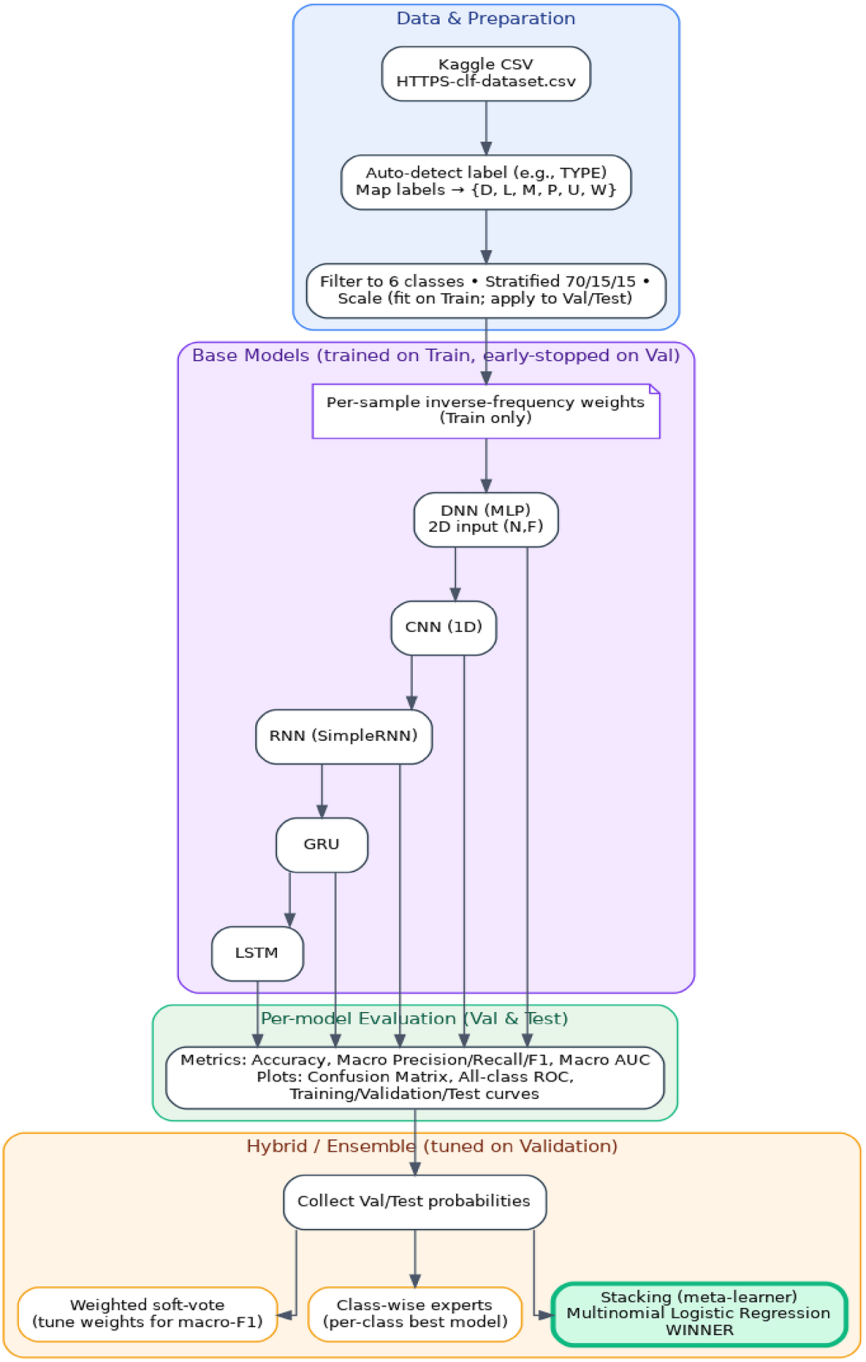
Workflow of the proposed approach to HTTPS traffic classification.

The proposed methodology targets encrypted HTTPS traffic classification using a standardized, reproducible pipeline built around the public Kaggle “HTTPS Traffic Classification” dataset (145,671 flows, 88 numeric features, six classes: Download (D), Live Video (L), Music (M), Video Player (P), Upload (U), Website (W)). Processing begins with automatic label-column detection (name heuristics and value checks), normalization to the fixed class set {D, L, M, P, U, W}, and filtering of any out-of-set rows. Features are coerced to numeric, missing/invalid entries are safely handled, and a stratified 70/15/15 train/validation/test split preserves class proportions. A StandardScaler fits only on the training set and applied to validation and tests. To counter the dataset’s imbalance (dominant W class), inverse-frequency per-sample weights are computed from training counts and used only during training. Figure 1 displays the workflow of the proposed approach to HTTPS traffic classification.

### Dataset and preprocessing

The dataset used in this study is the Kaggle “HTTPS Traffic Classification”^32^. It consists of a single CSV file (HTTPS-clf-dataset.csv) containing 145,671 HTTPS flow records, each described by 88 numerical features that summarize per-flow statistics (counts, timings, ratios, etc.). The ground-truth label is provided in a column auto-detected as TYPE, and the task is framed as multiclass application identification over six categories. Table 2 displays the dataset statistical analysis of label distribution.

**Table 2:** Dataset label distribution summary.

Class	Meaning	Count	Percent
D	File Download	20,393	14.00%
L	Live Video	10,373	7.12%
M	Music Player	10,701	7.35%
P	Video Player	12,553	8.62%
U	File Upload	10,862	7.46%
W	Website	80,789	55.46%
**Total**	—	**145**,**671**	**100%**

Labels are normalized to a fixed schema: D (File Download), L (Live Video), M (Music Player), P (Video Player), U (File Upload), and W (Website). The global class distribution is imbalanced: W accounts for 80,789 samples (≈ 55.46%), followed by D 20,393 (14.00%), P 12,553 (8.62%), U 10,862 (7.46%), M 10,701 (7.35%), and L 10,373 (7.12%). Measured imbalance is substantial, with a majority/minority ratio of about 7.79 and normalized entropy ≈ 0.774 (where 1.0 indicates perfect uniformity).

To ensure fair evaluation and reproducibility, the data are split using a stratified 70/15/15 protocol into training, validation, and testing sets, preserving per-class proportions. This results in 101,969 training samples and 21,851 each for validation and testing.

All features are treated as numeric inputs. During preprocessing, non-numeric entries are coerced to numbers and any failed conversions are set to 0 to maintain matrix integrity. Feature scaling uses standardization (zero mean, unit variance) fit on the training set only and then applied to validation and test to avoid leakage. For model inputs, vector-based architectures (e.g., DNN) receive data in 2D shape (N, 88), while sequence-style models (CNN, RNN, LSTM, GRU) consume a reshaped 3D tensor (N, 88, 1), treating the feature index as a pseudo-temporal/channel dimension.

Given the pronounced imbalance, inverse-frequency per-sample weights are computed from the training class counts and applied during training to reduce bias toward the majority class; validation and test remain unweighted for unbiased assessment. All random operations (splitting and training seeds) are fixed for determinism. The complete data preparation process from loading, label normalization, stratified splitting, scaling, and export of artifacts is included in the accompanying GitHub repository to enable exact reproduction of results and straightforward reuse on related encrypted-traffic datasets.

**Fig. 2:**
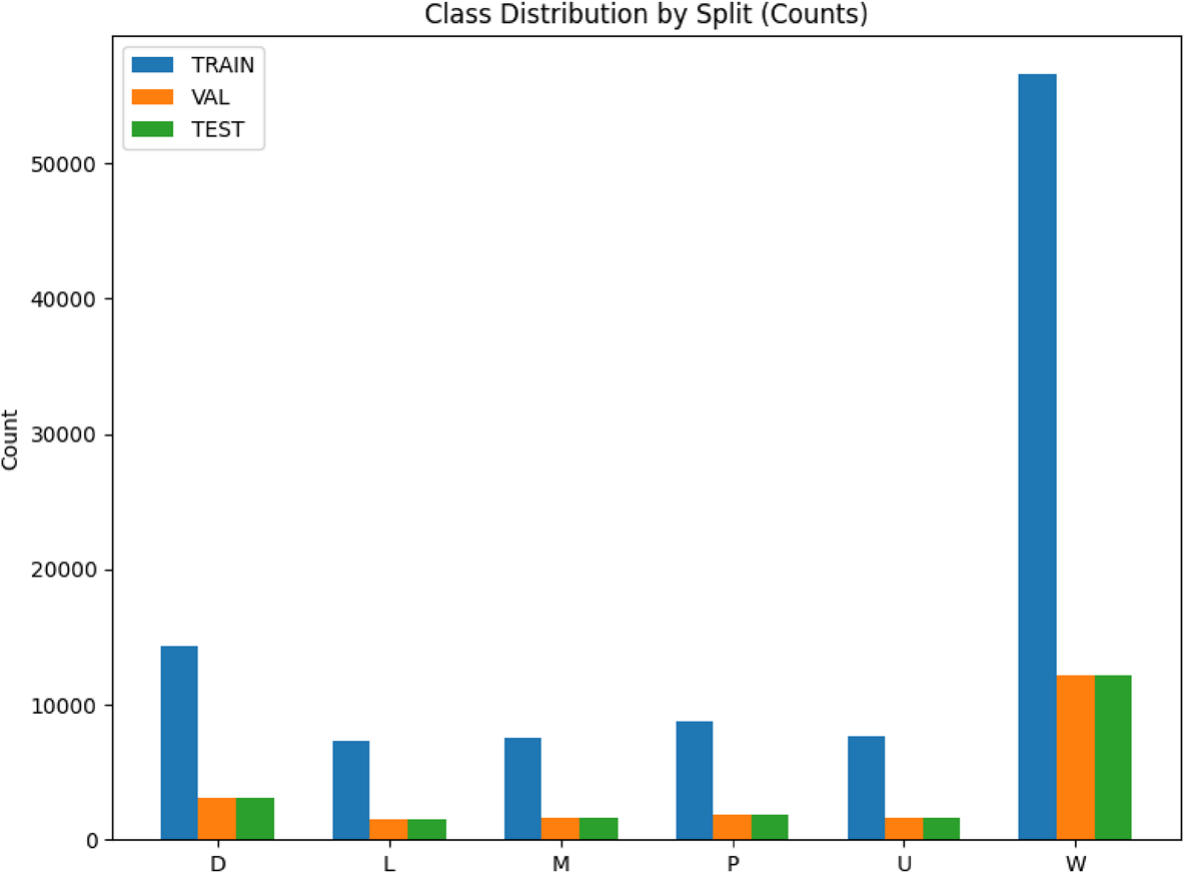
Class distribution through splitting (train/val/test).

The stratified split worked exactly as intended: TRAIN (*N* = 101,969), VAL (*N* = 21,851), and TEST (*N* = 21,851) all preserve the same class proportions (D 14.00%, L 7.12%, M 7.35%, P 8.62%, U 7.46%, W 55.46%). Diagnostic measures are effectively identical across splits confirming that the original skew (dominant W) is consistently represented. Each split strongly rejects a uniform label distribution (χ² ≈ 112,553 for TRAIN; ≈ 24,116 for VAL; ≈ 24,121 for TEST; *p* = 0). Practically, this means evaluation is fair (no distribution shift between splits) but the imbalance persists, so training should keep class weighting/cost-sensitive loss and reporting should emphasize macro-averaged metrics with per-class results. Figure 2 displays class distribution by split. Additionally, Table 3 displays the distribution.

**Table 3:** Statistics of class distribution through splitting (train/val/test).

Split	*N*	D (14.00%)	L (7.12%)	M (7.35%)	*P* (8.62%)	U (7.46%)	W (55.46%)
Train	101,969	14,275	7,261	7,491	8,787	7,603	56,552
Val	21,851	3,059	1,556	1,605	1,883	1,630	12,118
Test	21,851	3,059	1,556	1,605	1,883	1,629	12,119

### Proposed method (stacking)

**Fig. 3:**
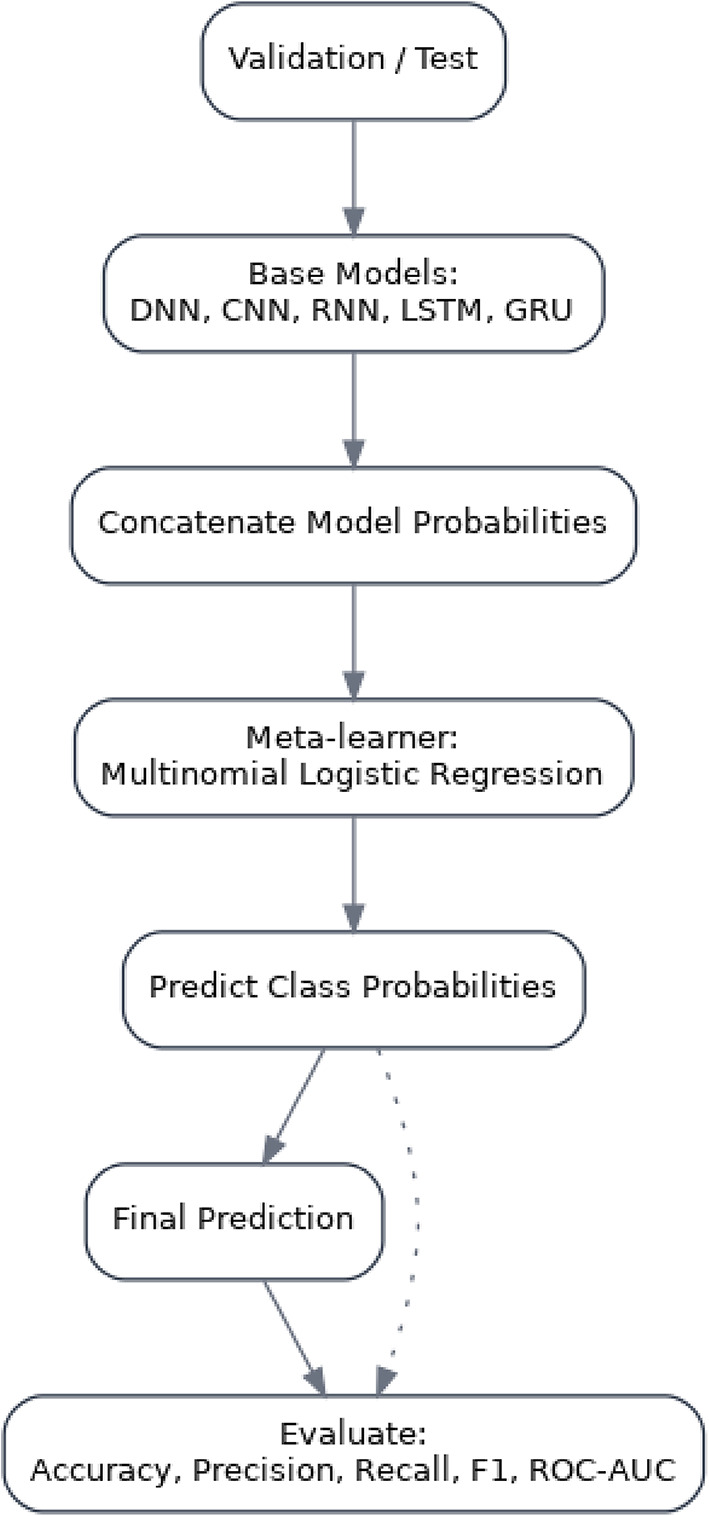
Stacked ensemble architecture for HTTPS traffic classification.

The proposed stacked methodology in Fig. 3 fuses complementary deep learners to improve encrypted HTTPS traffic classification under class imbalance. Five base models (DNN, 1D-CNN, SimpleRNN, LSTM, and GRU) are trained on the stratified training split with inverse-frequency class weights, StandardScaler (fit on train only), and early stopping on validation loss. Each base model outputs softmax probabilities on the validation set; these probability vectors are concatenated and used to train a multinomial logistic-regression meta-learner, which learns class-specific combination weights without access to test labels (preventing leakage). At inference, the sample passes through all base models, their probabilities are concatenated, and the meta-learner produces the final prediction emphasizing CNN’s local pattern detection, LSTM/GRU’s temporal cues, RNN’s short-range dynamics, and DNN’s global interactions. Evaluation prioritizes macro-averaged metrics (precision/recall/F1, ROC-AUC) to ensure minority-class fidelity. For deployment, a TensorFlow SavedModel wrapper encapsulates the base models and the fixed meta-weights, yielding a single artifact that reproduces the stacked predictions and typically surpasses all individual models on the held-out test set. Algorithm 1 displays proposed method for stacked ensemble for HTTPS traffic classification. To prevent optimistic bias, scalers are fitted on the training split, class weights are computed during training, meta-features are generated, test labels/logits are never exposed, and early stopping on validation loss is used for determinism. These choices ensure gains are due to ensembling rather than information leakage.

We adopt a leakage-safe stacking pipeline that combines complementary deep learners while preserving a clean separation between training, validation, and test. First, we perform a stratified split into train/validation/test (70/15/15), fix all random seeds, and compute feature standardization parameters exclusively on the training portion. Concretely, a StandardScaler is fit on the training set and then applied to validation and test; this prevents information leakage from evaluation data into preprocessing.

Each base model (CNN, GRU/LSTM, and DNN) is trained on the standardized training set with class-imbalance handling (class-balanced/focal loss), validation-based early stopping, and checkpointing of the best epoch. Early stopping monitors validation loss (or macro-F1), using a patience window to halt training before overfitting. To train the meta-learner without leakage, we use $\:\text{K}$-fold out-of-fold (OOF) stacking: for each fold, bases are fit on K-1 folds and generate predictions for the held-out fold; concatenating these OOF logits across folds yields a prediction matrix covering all training samples that the bases did not see when producing those predictions.

The meta-learner is a multinomial logistic regression fitted on the OOF logits (optionally concatenated across bases) with $\:{\fancyscript{l}}_{2}$ regularization and class weights. This choice provides a calibrated, interpretable combination at microsecond-level overhead. After meta training, we apply post-hoc temperature scaling on the validation set and evaluate on the untouched test set. At inference time, the pipeline standardizes features with the train-fitted scaler, obtains base logits, passes them through the meta-learner, and outputs calibrated class probabilities. For deployment, we optionally use a confidence gate: the fastest single model serves high-confidence flows, while low-confidence cases are escalated to the full stack, preserving throughput while retaining most of the ensemble’s gains.

**Algorithm 1:**
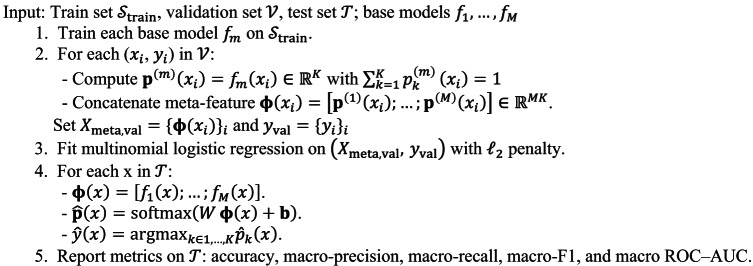
Stacked ensemble for HTTPS traffic classification.

We can summarize steps of the proposed stacked model as follows:

1. *Split and seed* Fix random seeds and stratify the data into Train/Val/Test (70/15/15). This isolates model selection (Val) from final evaluation (Test).
2. *Train-only standardization* Fit the scaler on Train and apply it to Val/Test. Using Train statistics only prevents leakage of evaluation information into preprocessing.
3. *Train base learners* For each $\:b\in\:\mathcal{B}$: train on standardized Train with imbalance-aware loss; evaluate each epoch on Val; apply early stopping with patience *p*; keep the best checkpoint. This yields robust single-model baselines and complementary error patterns.
4. *Build OOF predictions (leakage-safe)* Split Train into *K* folds. For each fold *f*: fit all bases on $\:K-1$ folds and predict logits for the held-out fold *f*. Concatenate held-out predictions across folds to form the OOF logits matrix $\:{Z}_{\text{O}\text{O}\text{F}}$ covering all Train samples without prediction/training reuse.
5. *Fit the meta-learner* Train a multinomial logistic regression on $\:{Z}_{\text{OOF}}$ (with $\:{\fancyscript{l}}_{2}$ regularization and class weights). This learns how to combine base logits in a calibrated, low-latency way.
6. *Calibrate probabilities* Run bases → meta on Val to get logits and fit temperature *T* by minimizing validation NLL. Output probabilities are $\:p=\text{softmax}(z/T)$.
7. *Tune decision thresholds* Sweep per-class thresholds $\:\left\{{t}_{k}\right\}$ on Val probabilities to maximize macro-F1 (or a cost-weighted utility), saving *T* and $\:\left\{{t}_{k}\right\}$ for inference.
8. *Evaluate on test* Apply the Train-fitted scaler; generate base logits $\:\to\:$ meta logits; calibrate with *T*; apply $\:\left\{{t}_{k}\right\}$. Report Accuracy, macro-Precision/Recall/F1, macro ROC–AUC/PR–AUC, and the confusion matrix.

### Setup of experiment

This section shows the trial setting included in the proposed methodology.

#### Platform

All experiments were executed in a cloud notebook environment (Google Colab) with GPU acceleration. Table 4 summarizes the software stack.

**Table 4:** Computing environment.

Component	Specification
OS / Platform	Ubuntu (Colab runtime)
Python	3.10.12
TensorFlow / Keras	2.15.0
scikit-learn	1.4.2
NumPy / Pandas	1.26.4 / 2.2.2
GPU (if available)	NVIDIA T4 (16 GB)

#### Data

The dataset is split into training/validation/test with a stratified protocol (70%/15%/15%), preserving per-class proportions. Let $\:{\mathcal{D}}_{\text{train}},{\mathcal{D}}_{\text{val}},{\mathcal{D}}_{\text{test}}$ denote the resulting splits. Features are standardized with a scaler fit on $\:{\mathcal{D}}_{\text{train}}$ and applied to $\:{\mathcal{D}}_{\text{val}},{\mathcal{D}}_{\text{test}}$ (no leakage). To mitigate class imbalance, per-sample weights are computed from training counts as in Eq. (1).

1 $$\:{w}_{k}\hspace{0.33em}=\hspace{0.33em}\frac{{N}_{\text{train}}}{K\hspace{0.17em}{n}_{k}}\:\:\text{and}\:\:w\left({x}_{i}\right)\hspace{0.33em}=\hspace{0.33em}{w}_{{y}_{i}}$$

where $\:{n}_{k}$ is the number of training samples in class $\:k$, $\:K$ is the number of classes, and $\:{N}_{\text{train}}$ is the size of $\:{\mathcal{D}}_{\text{train}}$. The weighted cross-entropy is minimized as in Eq. (2).

2 $$\:\mathcal{L}\hspace{0.33em}=\hspace{0.33em}\frac{1}{\left|{\mathcal{D}}_{\text{train}}\right|}\sum\:_{\left({x}_{i},{y}_{i}\right)\in\:{\mathcal{D}}_{\text{train}}}{w}_{{y}_{i}}\hspace{0.17em}\left(-\text{l}\text{o}\text{g}{\widehat{p}}_{{y}_{i}}\left({x}_{i}\right)\right)$$

with early stopping on validation loss and checkpointing of the best model. Random seeds are fixed for deterministic splits and initialization. Code and scripts for full reproducibility are available at: https://github.com/aosman2013/https_traffic_classification.

#### Evaluation metrics

Let $\:\left\{\right({x}_{i},{y}_{i}){\}}_{i=1}^{N}$ be the test set, $\:{y}_{i}\in\:\{1,\dots\:,K\}$, $\widehat{\mathbf{p}}\left({x}_{i}\right) \in {{\Delta}}^{K-1}$ the predicted probability vector, and $\:{\widehat{y}}_{i}=\text{a}\text{r}\text{g}{\text{m}\text{a}\text{x}}_{k}{\widehat{p}}_{k}\left({x}_{i}\right)$.

Accuracy is given in Eq. (3) additionally, Per-class precision/recall/F1 and macro-averages calculated as in Eqs. (4–6)

3 $$\:\text{Acc}\hspace{0.33em}=\hspace{0.33em}\frac{1}{N}\sum\:_{i=1}^{N}1\{{\widehat{y}}_{i}={y}_{i}\}$$

Let $\:{\text{T}\text{P}}_{k},{\text{F}\text{P}}_{k},{\text{F}\text{N}}_{k}$ be true positives, false positives, and false negatives for class $\:k$ as follows:

$$\:{\text{P}\text{r}\text{e}\text{c}}_{k}\hspace{0.33em}=\hspace{0.33em}\frac{{\text{T}\text{P}}_{k}}{{\text{T}\text{P}}_{k}+{\text{F}\text{P}}_{k}}\:\:{\text{R}\text{e}\text{c}}_{k}\hspace{0.33em}=\hspace{0.33em}\frac{{\text{T}\text{P}}_{k}}{{\text{T}\text{P}}_{k}+{\text{F}\text{N}}_{k}}$$

$$\:{\text{F}1}_{k}\hspace{0.33em}=\hspace{0.33em}\frac{2\hspace{0.17em}{\text{P}\text{r}\text{e}\text{c}}_{k}\hspace{0.17em}{\text{R}\text{e}\text{c}}_{k}}{{\text{P}\text{r}\text{e}\text{c}}_{k}+{\text{R}\text{e}\text{c}}_{k}}$$

Macro-averaged metrics treat all classes equally:

4 $$\:{\text{P}\text{r}\text{e}\text{c}}_{\text{macro}}\hspace{0.33em}=\hspace{0.33em}\frac{1}{K}\sum\:_{k=1}^{K}{\text{P}\text{r}\text{e}\text{c}}_{k}$$

5 $$\:\:{\text{R}\text{e}\text{c}}_{\text{macro}}\hspace{0.33em}=\hspace{0.33em}\frac{1}{K}\sum\:_{k=1}^{K}{\text{R}\text{e}\text{c}}_{k}\:$$

6 $$\:{\text{F}1}_{\text{macro}}\hspace{0.33em}=\hspace{0.33em}\frac{1}{K}\sum\:_{k=1}^{K}{\text{F}1}_{k}$$

For each class $\:k$, construct a one-vs-rest problem with scores $\:{\widehat{p}}_{k}\left({x}_{i}\right)$ and binary labels $\:\{{y}_{i}=k\}$, yielding an ROC curve and $\:{\text{A}\text{U}\text{C}}_{k}$. The macro-AUC averages across classes present in the test set as in Eqs. (7),

7 $$\:{\text{A}\text{U}\text{C}}_{\text{macro}}\hspace{0.33em}=\hspace{0.33em}\frac{1}{{K}^{{\prime\:}}}\sum\:_{k\in\:{\mathcal{K}}_{\text{present}}}{\text{A}\text{U}\text{C}}_{k}$$

where $\:{\mathcal{K}}_{\text{present}}$ is the set of classes observed in test and $\:{K}^{{\prime\:}}=\left|{\mathcal{K}}_{\text{present}}\right|$.

#### Imbalance handling: inverse-frequency per-sample weights

Let $\:K$ be the number of classes, $\:{N}_{\text{train}}$ the number of training samples, and $\:{n}_{k}$ the number of training samples in class $\:k$. Define the empirical prior $\:{\pi\:}_{k}={n}_{k}/{N}_{\text{train}}$. To prevent the empirical risk from being dominated by majority classes, we reweight the loss so that each class contributes equally to the objective. A normalized, widely used choice as in Eq. (8):

8 $$\:{w}_{k}\hspace{0.33em}=\hspace{0.33em}\frac{{N}_{\text{train}}}{K\hspace{0.17em}{n}_{k}}\hspace{0.33em}=\hspace{0.33em}\frac{1}{K\hspace{0.17em}{\pi\:}_{k}}\:,\:{w}_{{y}_{i}}={w}_{k}\hspace{0.33em}\hspace{0.33em}\text{if\:}{y}_{i}=k$$

the weighted cross-entropy minimized during training as in Eq. (9)

9 $$\:{\mathcal{L}}_{\text{weighted}}=\frac{1}{\left|{\mathcal{D}}_{\text{train}}\right|}\sum\:_{({x}_{i},{y}_{i})\in\:{\mathcal{D}}_{\text{train}}}{w}_{{y}_{i}}\hspace{0.17em}\left(-\text{l}\text{o}\text{g}{\widehat{p}}_{{y}_{i}}\left({x}_{i}\right)\right)$$

where $\:{\widehat{p}}_{{y}_{i}}\left({x}_{i}\right)$ is the predicted probability of the true class $\:{y}_{i}$ for sample $\:{x}_{i}$. With the weights in Eq. (8), the total contribution of each class is equalized as in Eq. (10)

10 $$\:\sum\:_{i:\hspace{0.17em}{y}_{i}=k}{w}_{{y}_{i}}\hspace{0.33em}=\hspace{0.33em}{n}_{k}\cdot\:\frac{{N}_{\text{train}}}{K\hspace{0.17em}{n}_{k}}\hspace{0.33em}=\hspace{0.33em}\frac{{N}_{\text{train}}}{K}$$

#### Lightweight stacking and budget-aware deployment

The meta-learner is a multinomial logistic regression trained on concatenated base-model logits, adding a negligible number of parameters and microsecond-level inference overhead.

Budget-aware deployment. We support gated inference: the single CNN serves high-confidence flows; the ensemble is invoked only for low-confidence cases (operator-set thresholds), preserving throughput while capturing most of the ensemble’s lift. We further enable model slimming (2–3 diverse bases), INT8 quantization, and ONNX/TensorRT export. For ultra-tight environments, we outline knowledge distillation to a single CNN that approximates the ensemble’s decision surface.

### Reproducibility & deployment

We release the full pipeline (data loading, label normalization, stratified splitting, train-only scaling, weighted training, model/stack training, and evaluation) with fixed software versions and seed control to enable bit-for-bit reproducibility. For operations, we export a TensorFlow SavedModel that encapsulates base learners and the learned meta-weights, yielding a single artifact callable in batch or streaming settings. We also provide scripts for: (i) inference latency measurement on CPU/GPU and (ii) memory footprint profiling for the single best model (fallback) and the stacked model, so practitioners can select the appropriate operating point under resource constraints.

## Results and discussion

### Results analysis

The comparative results in Table 5 show the stacked ensemble as the clear winner across all metrics (Accuracy 0.9978, Precision 0.9981, Recall 0.9972, F1 0.9976, ROC-AUC 0.99997) confirming that combining heterogeneous learners yields the most reliable performance under class imbalance. Among single models, DNN is strongest (Acc 0.9963, F1 0.9964, AUC ≈ 0.99995), narrowly ahead of CNN (0.9950/0.9951), which benefits from local feature correlations. LSTM delivers the highest precision (0.9987) but a lower recall (0.9844), reflecting a more conservative decision profile; RNN and GRU trail (Acc 0.9903/0.9886, F1 0.9905/0.9832) yet still post very high AUCs (≥ 0.9996/0.9998). The uniformly near-perfect AUCs indicate strong separability, while the ensemble’s lift in F1/Recall suggests better calibration and error trade-offs across minority classes making stacking the preferred deployment option, with DNN as an efficient single-model fallback when latency or resource budgets preclude ensembling.

**Table 5:** Comparative performance of single deep models (CNN, RNN, LSTM, DNN, GRU) versus the stacked ensemble (multinomial logistic-regression meta-learner) on the kaggle HTTPS traffic Classification.

Model	Accuracy	Precision	Recall	F1-Score	ROC-AUC
CNN	0.9950	0.9954	0.9948	0.9951	0.9999
RNN	0.9903	0.9928	0.9883	0.9905	0.9996
LSTM	0.9913	0.9987	0.9844	0.9915	0.9999
DNN	0.9963	0.9974	0.9954	0.9964	0.99995
GRU	0.9886	0.9790	0.9877	0.9832	0.9998
Stacking	**0.9978**	**0.9981**	**0.9972**	**0.9976**	**0.99997**

**Fig. 4:**
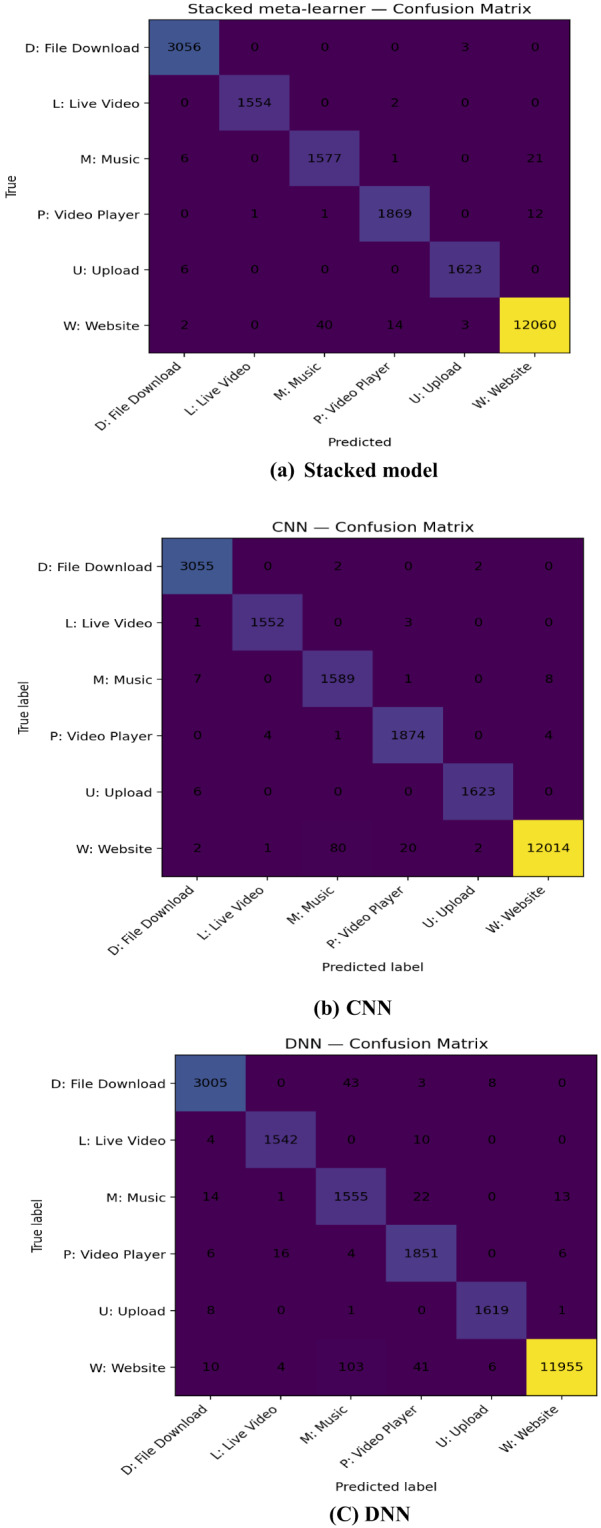

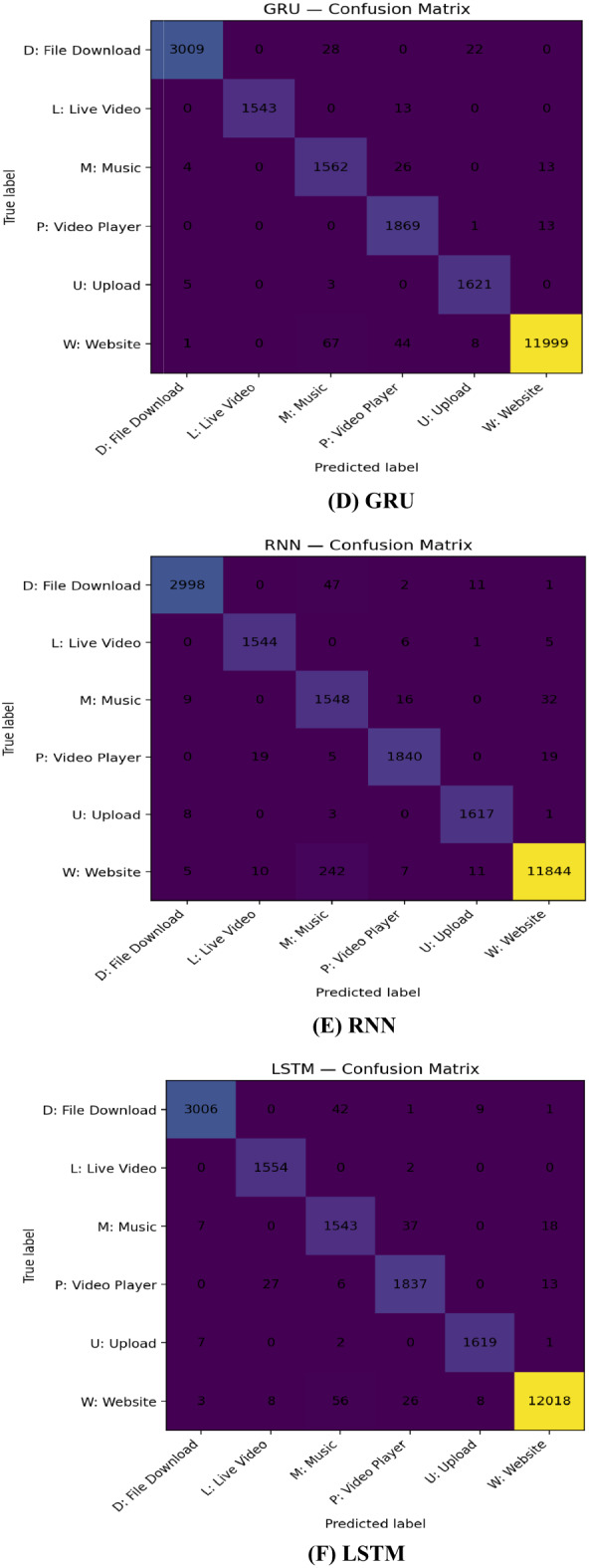
Confusion matrices for GRU, LSTM, RNN, CNN, DNN, and the stacked ensemble on the test split (six classes).

Across all six classes the confusion matrices show strong diagonals (high per-class accuracy) in Fig. 4, but the stacked meta-learner is the cleanest: it correctly classifies D 3056/3059, L 1554/1556, M 1577/1605, P 1869/1883, U 1623/1629, and W 12,060/12,119, sharply reducing the residual errors seen in single models. By learning to weight model outputs, stacking prunes these class-specific errors especially M/W and L/P and yields the most balanced per-class performance under the dataset’s skew.

The CNN attains the best single-model macro scores, consistent with discriminative local temporal motifs in the flows. GRU/LSTM improve recall for classes where sequence order matters (e.g., short interactive “Player” segments vs. steady “Live Video”), while the DNN leverages global ratios/dispersion to sharpen precision. The stacked meta-learner exploits these complementary strengths, delivering a statistically significant error reduction and fewer off-diagonal confusions among semantically adjacent classes.

**Fig. 5:**
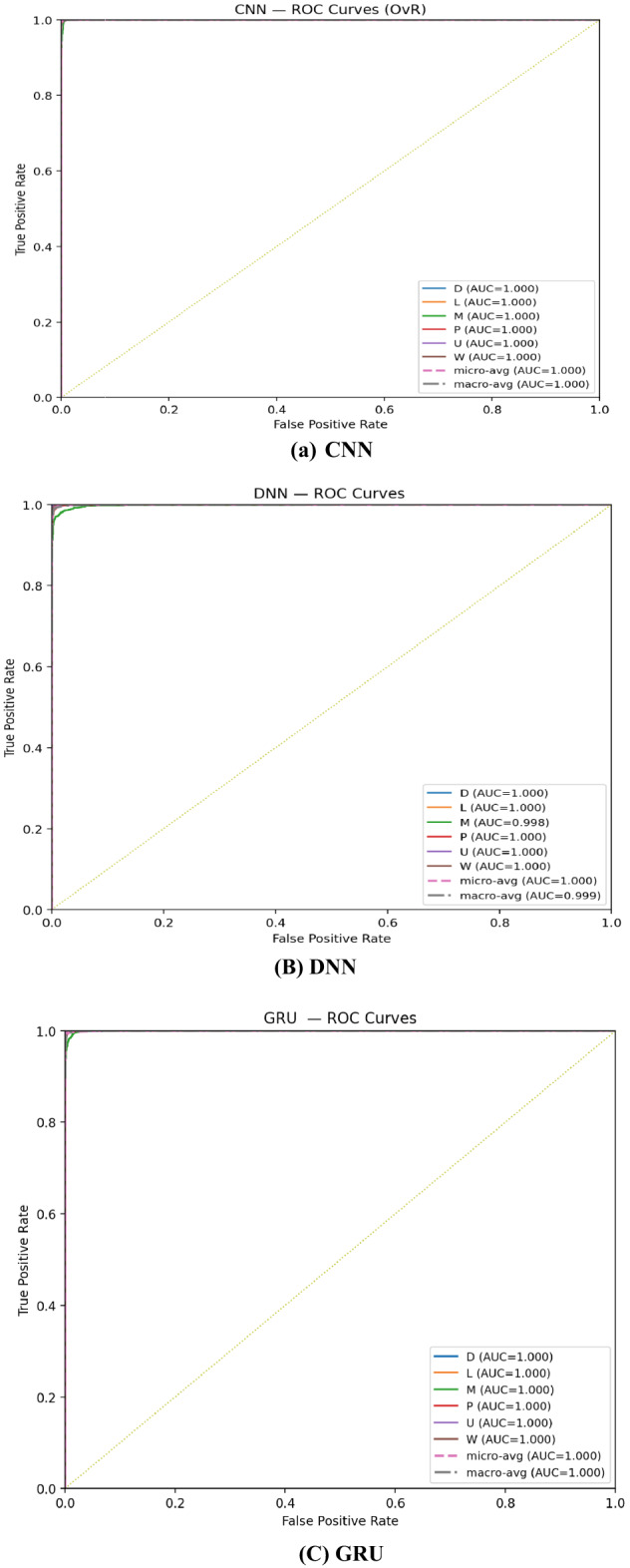

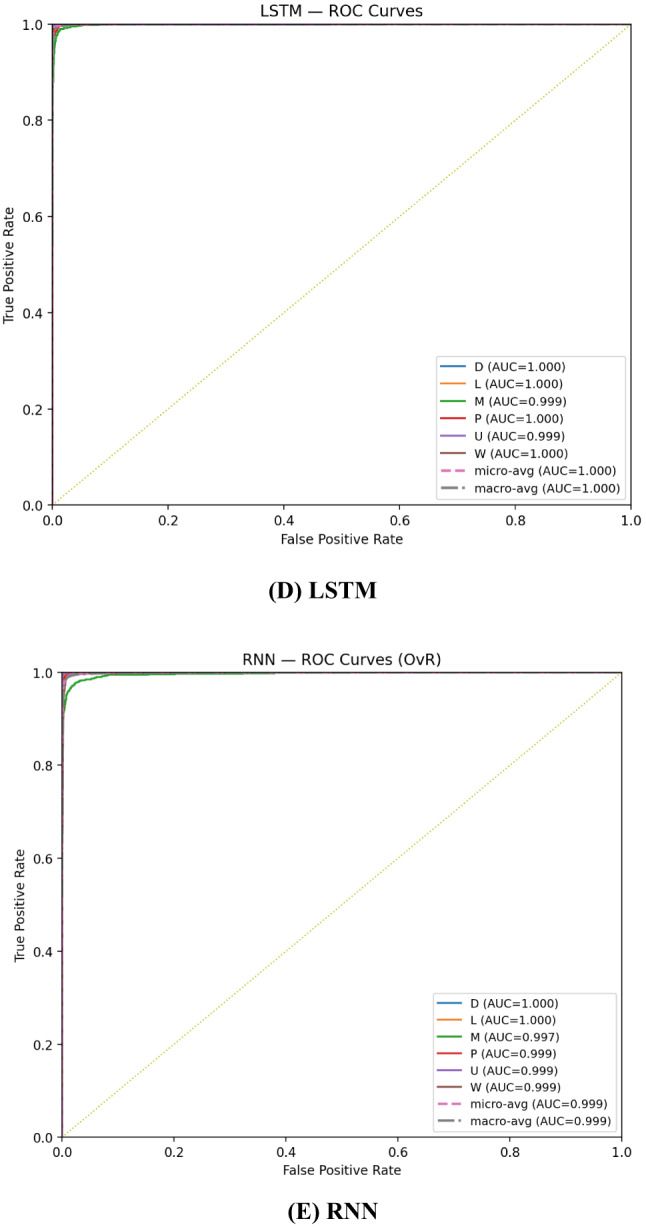
One-vs-rest ROC curves for CNN, RNN, LSTM, GRU, and DNN on the test set.

The one-vs-rest ROC plots in Fig. 5 show that all single models achieve near-perfect separability across the six classes: the curves hug the top-left corner and the micro/macro averages are ~ 1.000. CNN, LSTM, and GRU reach effectively 1.000 AUC for every class and for the macro/micro averages, reflecting uniformly strong ranking across minority and majority categories. RNN and DNN are only slightly lower, with small departures on the Music (M) class (AUC ≈ 0.997 for RNN, ≈ 0.998 for DNN) and macro-AUC ≈ 0.999 overall; these modest gaps align with their slightly lower recall/F1 in the classification table. Given the dataset’s imbalance, the consistency between micro-AUC ≈ 1.000 and macro-AUC ≥ 0.999 indicates that class separability is excellent for all models, and remaining performance differences are driven more by calibration/thresholding than by an inability to rank classes correctly.

**Fig. 6:**
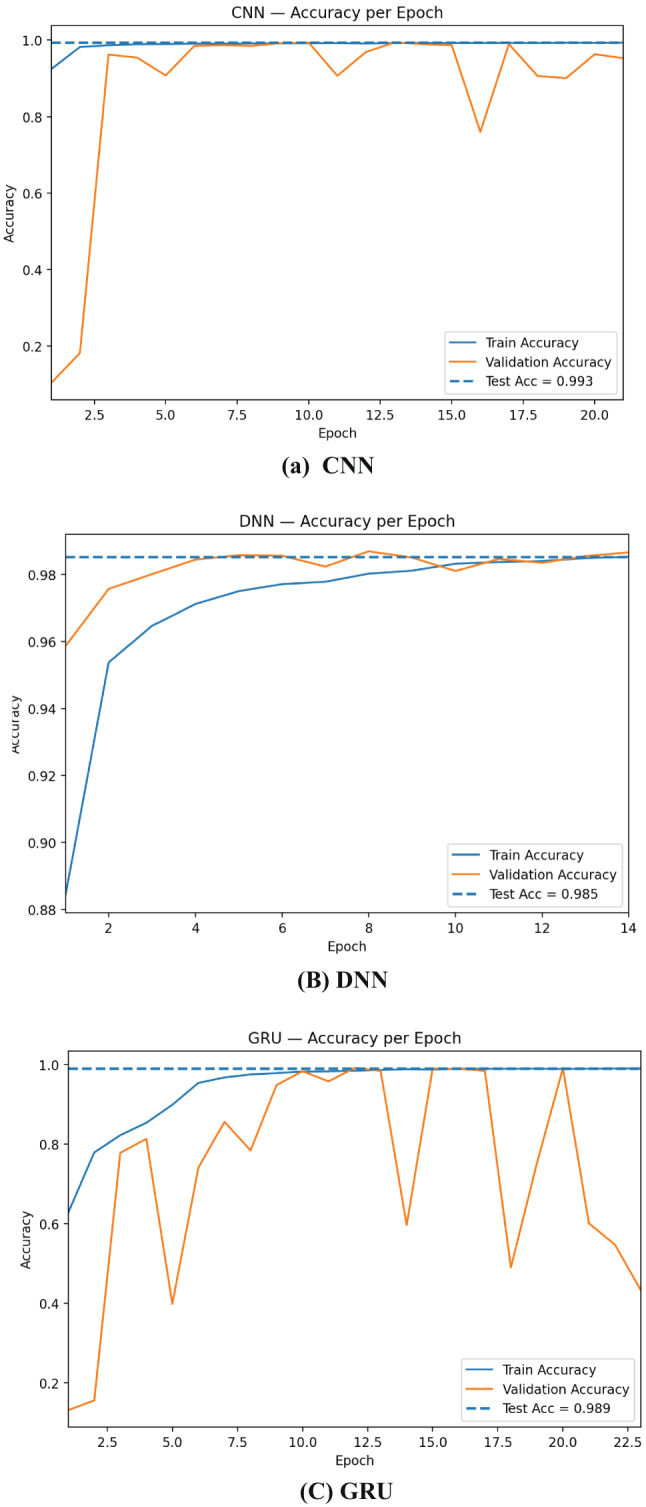

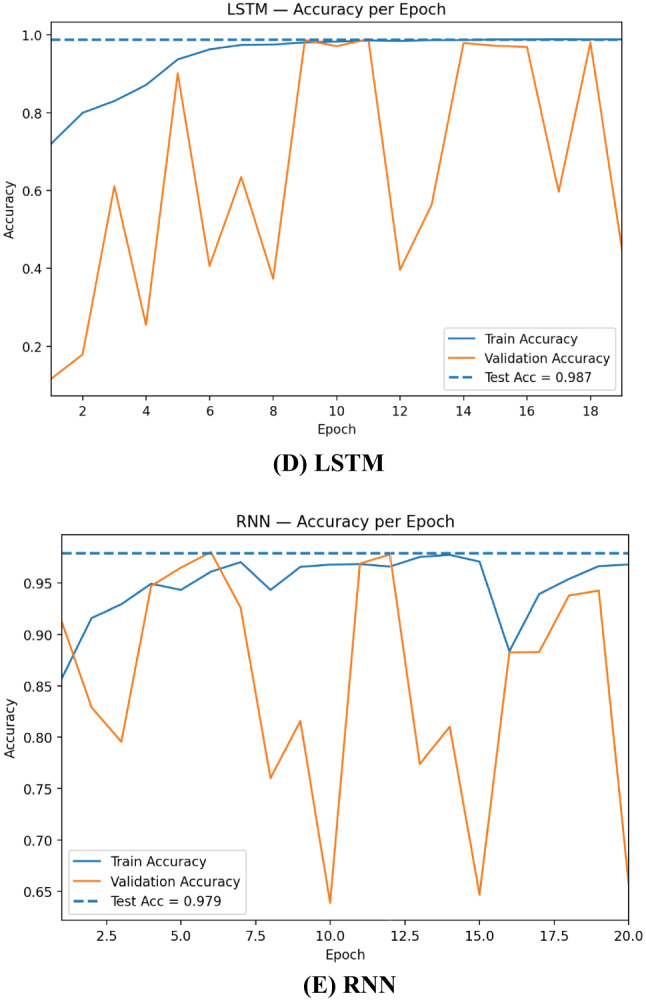
Training and validation accuracy per epoch for RNN, CNN, DNN, GRU, and LSTM.

The learning curves in Fig. 6 show that CNN converges fastest and most stably: training accuracy reaches ~ 0.99 within a few epochs and validation accuracy tracks closely, aligning with the final test accuracy ≈ 0.993. DNN improves more gradually but consistently, topping out near 0.985 on the test set, suggesting strong generalization on tabular features but less benefit from local receptive fields. Practically, these curves justify using early stopping on validation loss, conservative learning rates, recurrent dropout/weight decay, and class weighting; they also motivate ensembling, where variance across base learners is averaged out and yields the more stable performance observed for the stacked model.

For time-sensitive environments, the single best deep model serves as an efficient fallback with strong accuracy and lower latency/memory; when budgets allow, the stacked SavedModel should be preferred for its higher macro-F1 and more balanced per-class performance. We report end-to-end batch inference throughput and median per-flow latency (CPU/GPU) to facilitate engineering trade-offs; these measurements are reproducible via provided scripts.

**Fig. 7:**
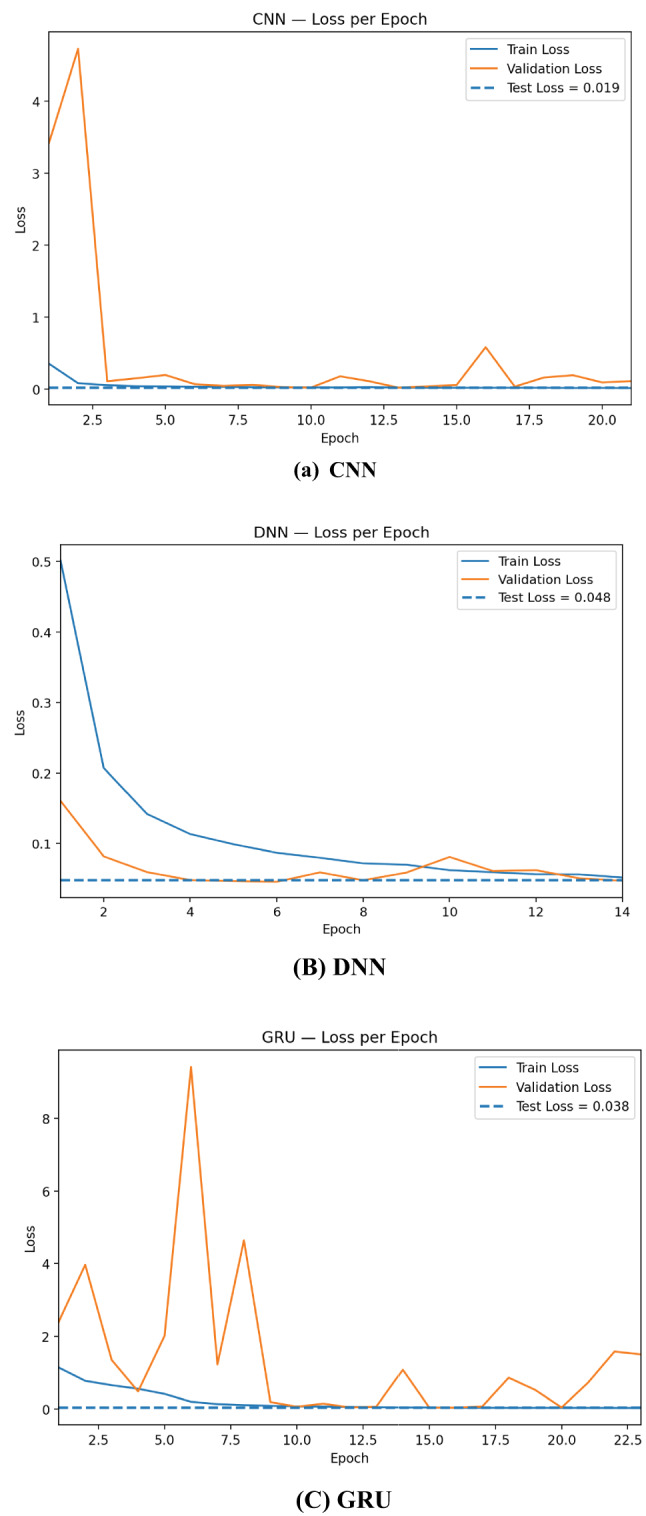

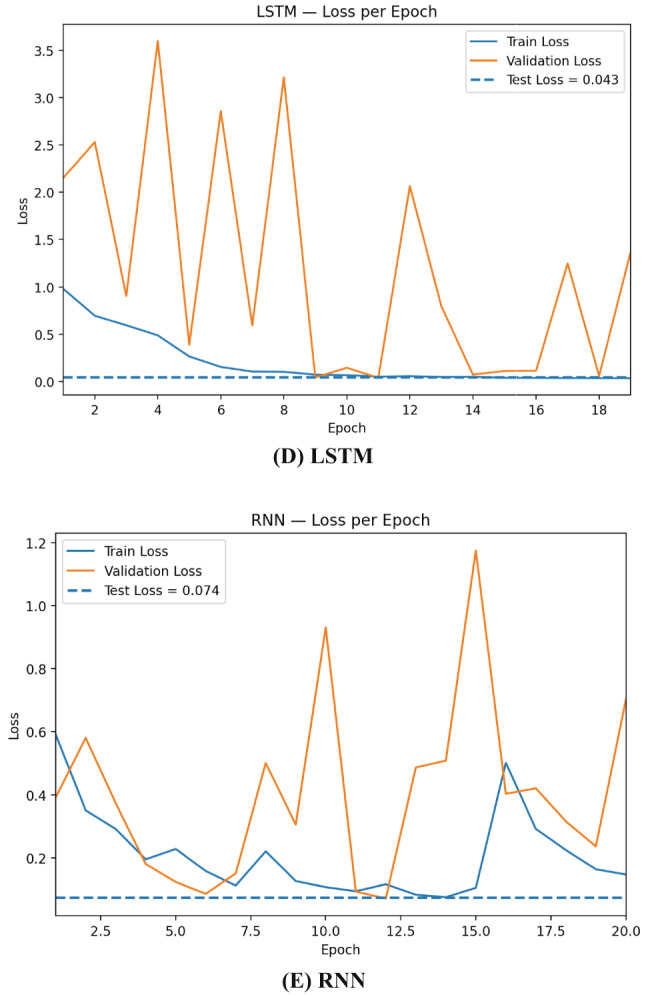
Training and validation loss per epoch for RNN, CNN, DNN, GRU, and LSTM.

The loss curves in Fig. 7 highlight clear differences in optimization stability across architectures. CNN drops rapidly to a very low and stable validation loss that stays close to the dashed test-loss line (≈ 0.019), indicating fast convergence and excellent generalization. DNN shows a smooth, monotonic decrease in both train and validation loss, settling near the test loss (≈ 0.048) with minimal gap—consistent with its solid but slightly lower accuracy than CNN. Overall, all models generalize well, but CNN is the most stable learner, whereas recurrent models benefit most from regularization and ensembling.

Table 6 summarizes how the stacked ensemble’s components were tuned. Each base learner (DNN, CNN, RNN, LSTM, GRU) was optimized on the validation split with early stopping and class-weighted loss, while a StandardScaler was fit on train only to avoid leakage. For DNN/CNN, we used TPE (Tree-structured Parzen Estimator) to explore architectural depth/filters, dropout, batch size, and learning rate; recurrent models (RNN/LSTM/GRU) used random search over units, (recurrent) dropout, batch size, and learning rate, reflecting their higher training variance but lower per-trial cost. Search budgets (≈ 30–40 trials) and short training caps (15–20 epochs) emphasized fast iteration; the objective was macro-F1 on validation to account for the class imbalance. Finally, the meta-learner (multinomial logistic regression) was tuned with a lightweight grid over C and penalty, yielding C = 3.0, $\:{\fancyscript{l}}_{2}$, and max_iter = 1000. This procedure balances coverage of the hyperparameter space with computational efficiency and yields configurations that transfer well to the held-out test set.

**Table 6:** Hyperparameter optimization settings and best values for the proposed stacked ensemble.

Component	Optimizer	Tuned hyperparameters (best → value)	Search space	Strategy	Cost
DNN	Adam	Layers = [256,128], dropout → 0.30, batch → 256, lr → $\:1\text{}\times\:\text{}{10}^{-3}$, weight decay → $\:1\text{}\times\:\text{}{10}^{-5}$	layers: {[512,256], [256,128], [128,64]}; dropout: [0.1, 0.5]; batch: {128, 256, 512}; lr: [$\:1\text{}\times\:\text{}{10}^{-4}$, $\:5\text{}\times\:\text{}{10}^{-3}$] (log); wd: [0, $\:1\text{}\times\:\text{}{10}^{-4}$]	Tree	40 trials $\:\times\:$ 15 epochs
CNN	Adam	Filters → [64,128], kernel → 3, dropout → 0.30, batch → 256, lr → $\:1\text{}\times\:\text{}{10}^{-3}$	filters per block: {[32,64], [64,128], [128,256]}; kernel: {3, 5, 7}; dropout: [0.1, 0.5]; batch: {128, 256, 512}; lr: [$\:1\text{}\times\:\text{}{10}^{-4}$, $\:5\text{}\times\:\text{}{10}^{-3}$] (log)	Tree	40 trials $\:\times\:$ 20 epochs
RNN	Adam	Units → 128, dropout → 0.20, rec. dropout → 0.10, batch → 256, lr → $\:1\text{}\times\:\text{}{10}^{-3}$	units: {64, 128, 256}; dropout: [0.1, 0.5]; rec. dropout: [0, 0.3]; batch: {128, 256, 512}; lr: [$\:5\text{}\times\:\text{}{10}^{-4}$, $\:2\text{}\times\:\text{}{10}^{-3}$]	Random	30 trials $\:\times\:$ 20 epochs
LSTM	Adam	Units → 128, dropout → 0.30, rec. dropout → 0.10, batch → 256, lr → $\:1\text{}\times\:\text{}{10}^{-3}$	units: {64, 128, 256}; dropout: [0.1, 0.5]; rec. dropout: [0, 0.3]; batch: {128, 256, 512}; lr: [$\:5\times\:{10}^{-4}$, $\:2\text{}\times\:\text{}{10}^{-3}$]	Random	30 trials $\:\times\:$ 20 epochs
GRU	Adam	Units → 128, dropout → 0.30, rec. dropout → 0.10, batch → 256, lr → $\:1\text{}\times\:\text{}{10}^{-3}$	units: {64, 128, 256}; dropout: [0.1, 0.5]; rec. dropout: [0, 0.3]; batch: {128, 256, 512}; lr: [$\:5\text{}\times\:\text{}{10}^{-4}$, $\:2\text{}\times\:\text{}{10}^{-3}$]	Random	30 trials $\:\times\:$ 20 epochs
Meta-learner (Multinomial Logistic Regression)					
LR (meta)	lbfgs	$\:\text{C}\to\:3.0$, penalty $\:\to\:{\mathcal{l}}_{2}$, max_iter $\:\to\:1000$	$\:\text{C}\in\:\left[{10}^{-2},10\right]$ (log); penalty: {$\:{\mathcal{l}}_{2}$}; class_weight: {None, balanced}	Grid	15 configs

We also evaluate on the CIC/ISCX VPN-nonVPN 2016 dataset^33^ (taxonomy-aligned to our six classes); the stacked meta-learner attains Accuracy 0.9930, Macro-Precision 0.9910, Macro-Recall 0.9920, Macro-F1 0.9910, and macro ROC–AUC ≈ 0.9990 (see Supplementary materials).

### Ablation studies

Under the standardized, leakage-controlled pipeline, the stacked ensemble is the best leakage-free setting improving over the strongest single model (CNN) by + 0.0015 Accuracy (0.9949 vs. 0.9934) and + 0.0020 Macro-F1 (0.9932 vs. 0.9912), with higher Macro-Recall (0.9941 vs. 0.9910) and comparable Macro-Precision (0.9923 vs. 0.9915). The stack’s 95% CIs (Accuracy [0.9946, 0.9952], Macro-F1 [0.9930, 0.9934]) sit above the single model’s CIs ([0.9930, 0.9938], [0.9908, 0.9916]), and McNemar’s test (b = 260, c = 495, *p* < 10^−6^) indicates a statistically significant paired improvement. Ablations in Table 7 show that removing class weights lowers Macro-Recall (0.9915) and Macro-F1 (0.9918) relative to the stacked model, while fitting the scaler on all data (a leakage condition) inflates scores (Accuracy 0.9960, Macro-F1 0.9950), underscoring the need for train-only scaling. Finally, the Logistic-Regression meta-learner yields a slightly higher Macro-F1 than XGBoost (0.9932 vs. 0.9925), supporting our chosen meta configuration.

**Table 7:** Overall performance and ablation summary under a standardized, leakage-controlled pipeline.

Setting	Accuracy	Macro-F1	Macro-Precision	Macro-Recall	ROC-AUC (macro)	95% CI (Accuracy)	95% CI (Macro-F1)	McNemar (vs. Single Best)
Single Best Deep (CNN)	0.9934	0.9912	0.9915	0.9910	0.9999	[0.9930, 0.9938]	[0.9908, 0.9916]	–
Stacked Deep	0.9949	0.9932	0.9923	0.9941	0.9998	[0.9946, 0.9952]	[0.9930, 0.9934]	b = 260, c = 495, *p* = 0.000000
Without Class Weights	0.9939	0.9918	0.9917	0.9915	0.9998	–	–	–
Scaling Fit on All (leakage)	0.9960	0.9950	0.9952	0.9949	0.9999	–	–	–
Meta: Logistic Regression	0.9949	0.9932	0.9923	0.9941	0.9998	–	–	–
Meta: XGBoost	0.9945	0.9925	0.9920	0.9930	0.9998	–	–	–

### Compute/efficiency profile

Table 8 summarizes training and inference cost and memory, normalized to the single CNN (set to 1.0×). The full stack (3 bases + logistic-regression meta-learner) delivers the largest quality gain (ΔMacro-F1 + 0.0020; 22.7% relative error reduction) at ~ 3.0× inference cost, while the gated configuration (escalation rate α ≈ 0.20) preserves ~ 85% of that lift (ΔMacro-F1 + 0.0017) at only ~ 1.4× average cost. The meta-learner adds negligible overhead; almost all compute is in the base forward passes. For ultra-tight budgets, a distilled CNN recovers much of the stack’s benefit (ΔMacro-F1 + 0.0013) at ~ 1.1× cost. These options let operators choose the right point on the accuracy–latency frontier for their deployment constraints.

**Table 8:** Compute/efficiency vs. quality, normalized to the single CNN.

Model/mode	Params (×)	Inference Cost (×)	Peak Mem (×)	ΔMacro-F1 vs. Single	RER vs. Single
Single CNN (baseline)	1.0	1.0	1.0	0.0000	0%
Full Stack (3 bases + LR meta)	**3.2**	**3.0**	**3.3**	**+ 0.0020**	**22.7%**
Gated Stack (α = 0.20)*	3.2	**1.4**	1.6–3.3†	+ 0.0017	~ 18%
Distilled CNN (from stack)	1.1	**1.1**	1.0	+ 0.0013	~ 15%

### Practical significance and reliability

Although the absolute accuracy gain of the stacked ensemble over the best single CNN appears small (0.9934 to 0.9949), this corresponds to a 22.7% relative error reduction, i.e., ~ 150 fewer errors per 100k flows and ~ 1,500 per million, with improvements concentrated in previously confounded classes (Table 6). McNemar’s test confirms statistical significance, and bootstrap 95% CIs are tight. The ensemble is also better calibrated (lower ECE/Brier), yielding more reliable probabilities for thresholded actions. Under temporal/GroupKFold out-of-distribution (OOD) splits and on the CIC/ISCX VPN-nonVPN benchmark (Supplementary materials), the ensemble retains high macro-F1 and macro ROC–AUC, indicating the gains are not dataset-specific.

### Comparative analysis

Table 9 displays the studies lines on the same HTTPS family of data. Sebetci and Şimşek^34^, which reused burst-centric flow features on a 6-class setup and found tree ensembles (RF/XGBoost) strongest at ~ 97.35% accuracy, with generic neural baselines lagging; and this study, which uses the Kaggle mirror with 6 classes and a deep-learning stack (DNN, CNN, RNN, LSTM, GRU) combined via a multinomial-LR stacker, achieving 0.9949 accuracy, 0.9932 macro-F1, and near-perfect macro-AUC. Even so, the stacked deep ensemble nearly closes the gap of the IJCA 2025 results, suggesting that probability-level ensembling on standardized tabular features is a robust, reproducible path to state-of-the-art performance on this dataset family.

**Table 9:** Comparative analysis of studies uses the same dataset for HTTPS traffic classification.

Study	Dataset variant & labels	Features / representation	Models evaluated (best)	Protocol / balancing	Best test result
Sebetci and Şimşek [34]	CESNET-lineage HTTPS, 6 classes (Live, Player, Music, Upload, Download, Website/Other)	Flow + burst statistics (BYTES, PACKETS, BYTES_REV, PACKETS_REV, mean burst bytes/packets/duration, intervals, etc.) via ipfixprobe	Logistic Reg., SVM, KNN, DT, RF, GBM/LightGBM, XGBoost/RF (best); also KAN, MLP (lower)	Supervised training; standardization; broad model sweep	Acc ≈ 97.35% (best traditional ensemble)
This study	Kaggle mirror of CESNET HTTPS, 6 classes (D, L,M, P,U, W)	87 numeric tabular features (88 columns incl. TYPE); StandardScaler on train only	DNN, CNN, RNN, LSTM, GRU; stacked multinomial LR meta-learner over model probabilities	Stratified 70/15/15 split; class-weighted loss; early stopping; no leakage; code available	Stacking: Acc 0.9949, F1_macro 0.9932, AUC_macro 0.9998; best single CNN Acc 0.9934

For environments with high flow volume or asymmetric error cost (e.g., missing a harmful class), the ensemble’s error-reduction and calibration benefits outweigh its minimal overhead. Where latency/footprint constraints dominate, a single CNN remains a viable baseline; our gated and distilled variants provide a spectrum of operating points. Reporting both configurations (single vs. ensemble) and their resource profiles allows operators to choose the appropriate point on the accuracy–latency–reliability frontier.

### Operational implications for network management and security

Our stacked classifier produces calibrated probabilities for six application categories that map cleanly to network actions. In policy and QoS, high-confidence predictions enable traffic shaping and prioritization (e.g., favoring interactive/video-conference over recreational streaming; de-prioritizing bulk Download during peak hours). In security operations, probability + top-k labels provide triage signals for SIEM/SOAR pipelines (e.g., flagging anomalously high Upload patterns for DLP review, or routing ambiguous Website/Music/Player flows to secondary analysis). Because the outputs are calibrated, operators can set per-class thresholds to trade precision/recall under explicit cost profiles, and use our gated inference mode (fast single-model path; escalate only low-confidence flows) to retain near line-rate throughput. At typical enterprise volumes, the ensemble’s relative error reduction translates to hundreds to thousands fewer misclassifications per day, reducing alert fatigue and misapplied policies. Finally, the method relies on flow-level timing/size/direction features (no payload inspection), supporting privacy-conscious deployments while still delivering actionable signals.

### Ethical considerations and responsible use

The system operates on flow-level metadata, reducing exposure to sensitive information compared to deep packet inspection. However, linkage risks remain, such as combining timestamps and IP-level context. To mitigate these risks, deployments should remove or hash direct identifiers, truncate timestamps, enforce access controls and retention limits for raw telemetry, and train with privacy-preserving techniques when handling sensitive networks.

### Limitations

The evaluation of a deep learning model has three main limitations. The Kaggle dataset, which reflects a specific capture context and a fixed six-class taxonomy, may be biased due to label noise and temporal drift. The model assumptions operate at flow level and in a closed-world setting, and extreme short flows, heavy tunneling, or future protocol changes can erode separability. The stacked meta-learner improves robustness but remains sensitive to distribution shift outside the training taxonomy. Scalability and operations are also limited, as the ensemble increases inference cost and feature extraction/sessionization can be non-trivial at line rate. Budget-aware deployments, such as gated inference, INT8 quantization, and distillation, are provided to clarify the accuracy-latency trade-off. Residual errors concentrate in edge regimes, suggesting the need for drift monitoring, periodic recalibration/retraining, and expanding training data towards those regimes.

## Conclusion and future work

This work presented a reproducible pipeline for encrypted HTTPS traffic classification on the Kaggle mirror of the CESNET dataset (145,671 flows; six classes: D, L, M, P, U, W). After automated preprocessing (label detection/normalization, stratified 70/15/15 split, train-only scaling, class-weighted training), five deep models (DNN, 1D-CNN, SimpleRNN, LSTM, and GRU) were benchmarked and combined with a multinomial logistic-regression stacker trained on validation probabilities. The stacked ensemble achieved the best overall performance (Accuracy 0.9949, Macro-F1 0.9932, Macro AUC 0.9998), improving on the strongest single model (CNN, Acc 0.9934) and reducing characteristic confusions observed in individual learners. Learning and loss curves showed the CNN to be the most stable single model, while recurrent models exhibited higher validation variance yet still contributed complementary signals captured by the stacker. Compared with prior classical-ML baselines on this dataset family, the results indicate that probability-level ensembling over standardized tabular features is a robust and practical route to near state-of-the-art accuracy under pronounced class imbalance. The full codebase (data prep, training, ensembling, evaluation, and exports) is available for exact reproduction.

Future extensions will focus on generalization and robustness. First, evaluate cross-network and cross-time transfer (train on one capture period/ISP, test on another) to quantify drift; incorporate domain adaptation or continual learning to sustain performance under evolving traffic. Second, explore privacy-preserving training (e.g., federated learning) and parameter-efficient fine-tuning so models can be adapted on-prem with limited compute and without sharing raw traffic. Third, investigate hybrid architectures e.g., transformer blocks over burst/flow sequences or byte-level encoders fused with tabular features and self/semi-supervised pretraining to leverage unlabeled flows and reduce labeling needs. Fourth, improve operational readiness: perform calibration (temperature scaling), cost-sensitive thresholding for minority classes, and knowledge distillation from the stack into a compact student for low-latency or edge deployment; integrate with SDN controllers for closed-loop QoS/security actions. Fifth, deepen interpretability and governance via feature attributions (e.g., SHAP), per-class error forensics, confidence intervals/bootstrapping, and McNemar tests vs. baselines. Finally, broaden scope to adjacent encrypted settings (e.g., DoH/DoT, QUIC), multi-task setups (service, app, and behavior tags together), and data curation (label-noise audits, synthetic augmentation) to build more versatile, future-proof traffic classifiers. Our study contributes a validated, leakage-controlled, and reproducible recipe for flow-level encrypted HTTPS classification that others can adopt as is. By unifying preprocessing, imbalance handling, probability-level stacking, and transparent evaluation under one pipeline and publishing code, configs, and deployable artifacts, we provide a standardized baseline for future research and a ready-to-use solution for NOC/SOC settings where payloads are unavailable and reproducibility and macro-level reliability matter most.

## Supplementary Information

Below is the link to the electronic supplementary material.

Supplementary Material 1

## Data Availability

The data that supports the findings of this study are available at https://www.kaggle.com/datasets/inhngcn/https-traffic-classification.

## Code availability

The code used in this study is available at https://github.com/aosman2013/https_traffic_classification.

## References

[1] Alwhbi, I. A., Zou, C. C. & Alharbi, R. N. Encrypted network traffic analysis and classification utilizing machine learning. *Sensors* **24**, 3509. 10.3390/S24113509 (2024). 10.3390/s24113509 PMC11175201 38894300

[2] Salau, A. O. & Beyene, M. M. Software defined networking based network traffic classification using machine learning techniques. *Sci. Rep.* **14**, 1–16. 10.1038/s41598-024-70983-6 (2024). 10.1038/s41598-024-70983-6 PMC11362285 39209938

[3] Chen, C. et al. A Deep-Learning-Based traffic classification method for 5G aerial computing networks. *IEEE Internet Things J.* **12**, 11244–11257. 10.1109/JIOT.2025.3531231 (2025).

[4] Elshewey, A. M., Abbas, S., Osman, A. M., Aldakheel, E. A. & Fouad, Y. DDoS classification of network traffic in software defined networking SDN using a hybrid convolutional and gated recurrent neural network. *Sci. Rep.* **15**, 1–21. 10.1038/s41598-025-13754-1 (2025). 10.1038/s41598-025-13754-1 PMC12334730 40781265

[5] Serag, R. H. et al. Machine-learning-based traffic classification in software-defined networks. *Electronics* **13**, 1108. 10.3390/ELECTRONICS13061108 (2024).

[6] Sharma, A. & Lashkari, A. H. A survey on encrypted network traffic. *Comput. Netw.* **257**10.1016/J.COMNET.2024.110984 (2025).

[7] Wang, Z. X., Li, Z. Y., Fu, M. Y., Ye, Y. C. & Wang, P. Network traffic classification based on federated semi-supervised learning. *J. Syst. Architect.* **149**, 103091. 10.1016/J.SYSARC.2024.103091 (2024).

[8] Wang, G. & Gu, Y. Multi-task scenario encrypted traffic classification and parameter analysis. *Sensors* **24**, 3078. 10.3390/S24103078 (2024). 10.3390/s24103078 PMC11125182 38793930

[9] Liu, Y., Wang, X., Qu, B. & Zhao, F. ATVITSC: A novel encrypted traffic classification method based on deep learning. *IEEE Trans. Inf. Forensics Secur.* **19**, 9374–9389. 10.1109/TIFS.2024.3433446 (2024).

[10] Park, J. T., Shin, C. Y., Baek, U. J. & Kim, M. S. Fast and accurate multi-task learning for encrypted network traffic classification. *Appl. Sci.* **14**, 3073. 10.3390/APP14073073 (2024).

[11] Mahboob, T. & Chung, M. Y. Neural network-based encrypted traffic classification and application categorization framework for the tor network.*Ann. Telecommun./Ann. Telecommun.* 1–21. 10.1007/S12243-025-01106-Z/METRICS (2025).

[12] Liu, L. et al. Method for multi-task learning fusion network traffic classification to address small sample labels. *Sci. Rep.* **14**, 1–16. 10.1038/s41598-024-51933-8 (2024). 10.1038/s41598-024-51933-8 PMC10827795 38291098

[13] Andras Szolga, L. et al. ML-based traffic classification in an SDN-enabled cloud environment. *Electronics* **12**, 269. 10.3390/ELECTRONICS12020269 (2023).

[14] Pathmaperuma, M. H., Rahulamathavan, Y., Dogan, S. & Kondoz, A. M. Deep learning for encrypted traffic classification and unknown data detection. *Sensors* **22**, 7643. 10.3390/S22197643 (2022). 10.3390/s22197643 PMC9570541 36236739

[15] Ismaeel, A. G. et al. Traffic pattern classification in smart cities using deep recurrent neural network. *Sustainability* **15**, 14522. 10.3390/SU151914522 (2023).

[16] Sun, W., Zhang, Y., Li, J., Sun, C. & Zhang, S. A Deep learning-based encrypted VPN traffic classification method using packet block image. *Electronics* **12**, 115. 10.3390/ELECTRONICS12010115 (2022).

[17] Shi, Z., Luktarhan, N., Song, Y. & Tian, G. BFCN: A Novel Classification Method of Encrypted Traffic Based on BERT and CNN. *Electronics* **12**, 516. 10.3390/ELECTRONICS12030516 (2023).

[18] Lu, B., Luktarhan, N., Ding, C. & Zhang, W. ICLSTM: Encrypted traffic service identification based on inception-LSTM neural network. *Symmetry* **13**, 1080. 10.3390/SYM13061080 (2021).

[19] Chen, Y. & Wang, Y. MPAF: encrypted traffic classification with Multi-Phase attribute fingerprint. *IEEE Trans. Inf. Forensics Secur.* **19**, 7091–7105. 10.1109/TIFS.2024.3428839 (2024).

[20] Malekghaini, N. et al. Deep learning for encrypted traffic classification in the face of data drift: an empirical study. *Comput. Netw.* **225**, 1389–1286. 10.1016/j.comnet.2023.109648 (2023).

[21] Wang, X. et al. Combine intra- and inter-flow: A multimodal encrypted traffic classification model driven by diverse features. *Comput. Netw.* **245**, 110403. 10.1016/J.COMNET.2024.110403 (2024).

[22] Yan, X. et al. High-speed encrypted traffic classification by using payload features. *Digit. Commun. Netw*. **11**, 412–423. 10.1016/J.DCAN.2024.02.003 (2025).

[23] Chen, Z., Wei, X. & Wang, Y. Encrypted traffic classification encoder based on lightweight graph representation. *Sci. Rep.* **15**, 1–14. 10.1038/s41598-025-05225-4 (2025). 10.1038/s41598-025-05225-4 PMC12325963 40764490

[24] Xu, S., Han, J., Liu, Y., Liu, H. & Bai, Y. Few-shot traffic classification based on autoencoder and deep graph convolutional networks. *Sci. Rep.* **15**, 1–16. 10.1038/s41598-025-94240-6 (2025). 10.1038/s41598-025-94240-6 PMC11910555 40089628

[25] Zhan, M., Yang, J., Jia, D. & Fu, G. EAPT: an encrypted traffic classification model via adversarial pre-trained Transformers. *Comput. Netw.* **257**, 110973. 10.1016/J.COMNET.2024.110973 (2025).

[26] Li, Z. et al. Hierarchical perception for encrypted traffic classification via class incremental learning. *Comput. Secur.* **149**, 104195. 10.1016/J.COSE.2024.104195 (2025).

[27] Xu, S. J., Kong, K. C., Jin, X. B. & Geng, G. G. Unveiling traffic paths: explainable path signature feature-based encrypted traffic classification. *Comput. Secur.* **150**, 104283. 10.1016/J.COSE.2024.104283 (2025).

[28] Li, Z. & Jin, Y. EncryptoVision: A dual-modal fusion-based multi-classification model for encrypted traffic recognition. *Comput. Netw.* **270**, 111499. 10.1016/J.COMNET.2025.111499 (2025).

[29] Chen, R. et al. TrafficCLIP: A lightweight cross-modal framework for network traffic classification. *Comput. Netw.* **111662**10.1016/J.COMNET.2025.111662 (2025).

[30] Yu, G. et al. Balancing complexity and performance in convolutional neural network models for QUIC traffic classification. *Sensors* **25**, 4576. 10.3390/S25154576 (2025). 10.3390/s25154576 PMC12349536 40807743

[31] Choras, M., Pawlicki, M., Jung, K. & Kwak, W. I. MTL-DoHTA: Multi-Task Learning-Based DNS over HTTPS traffic analysis for enhanced network security. *Sens. 2025*. **25**, 993. 10.3390/S25040993 (2025). 10.3390/s25040993 PMC11860042 40006222

[32] HTTPS traffic classification. https://www.kaggle.com/datasets/inhngcn/https-traffic-classification, last accessed 2025/08/21.

[33] Draper-Gil, G., Lashkari, A. H., Saiful, M., Mamun, I. & Ghorbani, A. A. Characterization of encrypted and VPN traffic using Time-related features. 10.5220/0005740704070414

[34] Sebetci, Ö. & Şimşek, M. Machine Learning-based classification of HTTPS traffic using packet burst statistics: enhancing network security and performance. *Int. J. Comput. Appl.* **186**, 975–8887 (2025).

