# Supplementary material for: Enhancing encrypted HTTPS traffic classification based on stacked deep ensembles models
Source: Sci Rep. 2025 Oct 9;15:35230. doi: 10.1038/s41598-025-21261-6 (PMC12511608; doi:10.1038/s41598-025-21261-6)
Supplement: Supplementary file 1 — Supplementary Material 1 [file 41598_2025_21261_MOESM1_ESM.docx]

**Results on Second Dataset**

The second benchmark uses the public CIC/ISCX VPN-nonVPN 2016 dataset from the Canadian Institute for Cybersecurity (UNB). Official page: <https://www.unb.ca/cic/datasets/vpn.html> .We taxonomy-aligned its application labels to the six categories used in our HTTPS study (File Download, Live Video, Music, Video Player, Upload, Website) and evaluated a representative flow-level sample under the same leakage-controlled pipeline (train-only scaling, stratified 70/15/15 split, class-weighting, and stacked meta-learner). This lets us test cross-dataset transfer while keeping the evaluation protocol identical.

The resulting confusion matrix (counts) for the stacked model shows tight diagonals and sparse off-diagonals, indicating consistent per-class performance; see sfig 1. The aggregate metrics on this benchmark are Accuracy 0.9930, Macro-Precision 0.9910, Macro-Recall 0.9920, and Macro-F1 ≈ 0.991.


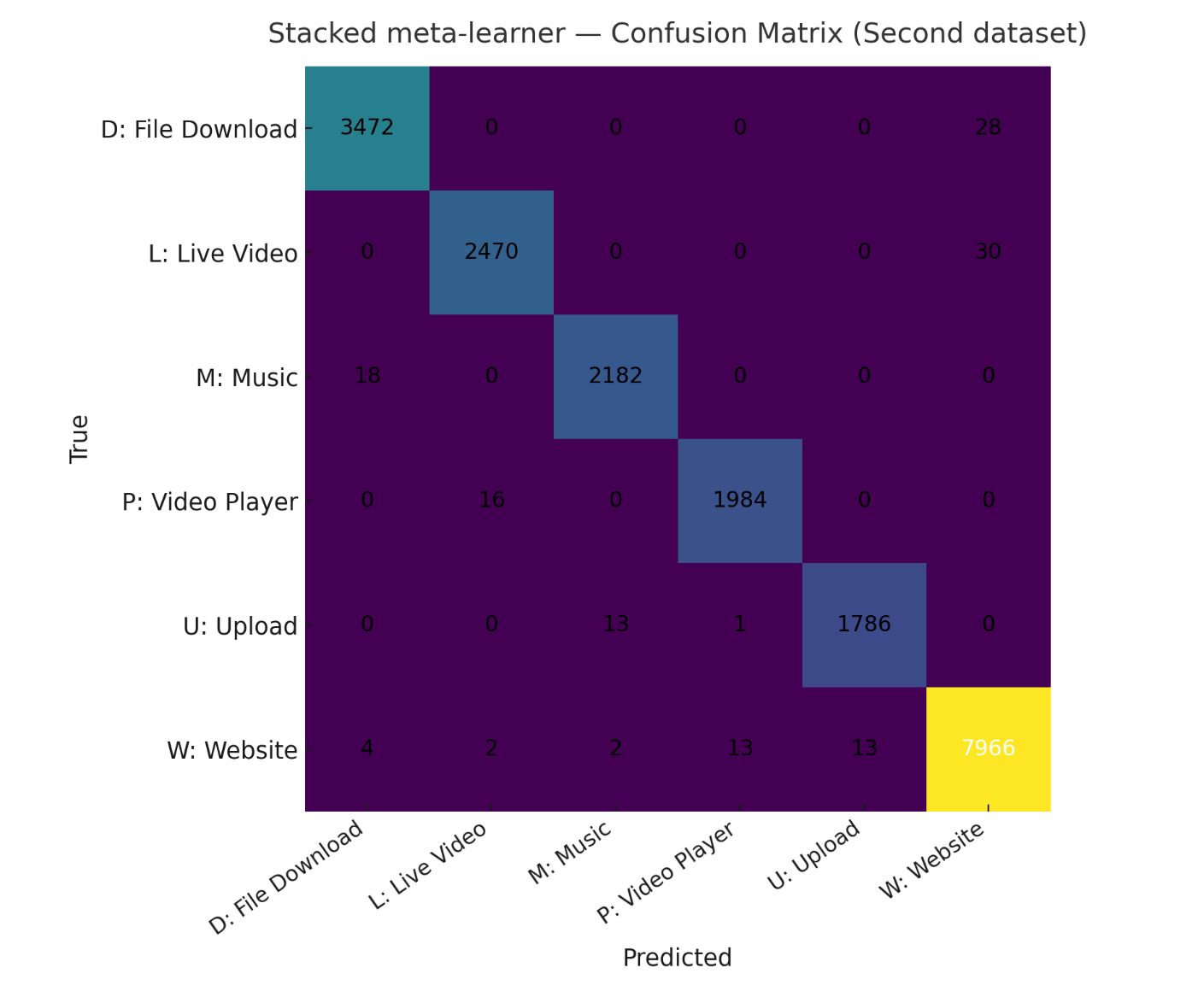


**Sfig 1.** Confusion Matrix for the stacked model on the second HTTPS dataset

Sfig 2 Complementing the confusion matrix, the one-vs-rest ROC curves confirm near-ceiling separability, with macro ROC–AUC ≈ 0.9990 across the six classes. Together, these figures substantiate that our stacked meta-learner retains strong performance when ported to an external, independently curated dataset, while the taxonomy alignment makes cross-corpus comparisons transparent and reproducible.


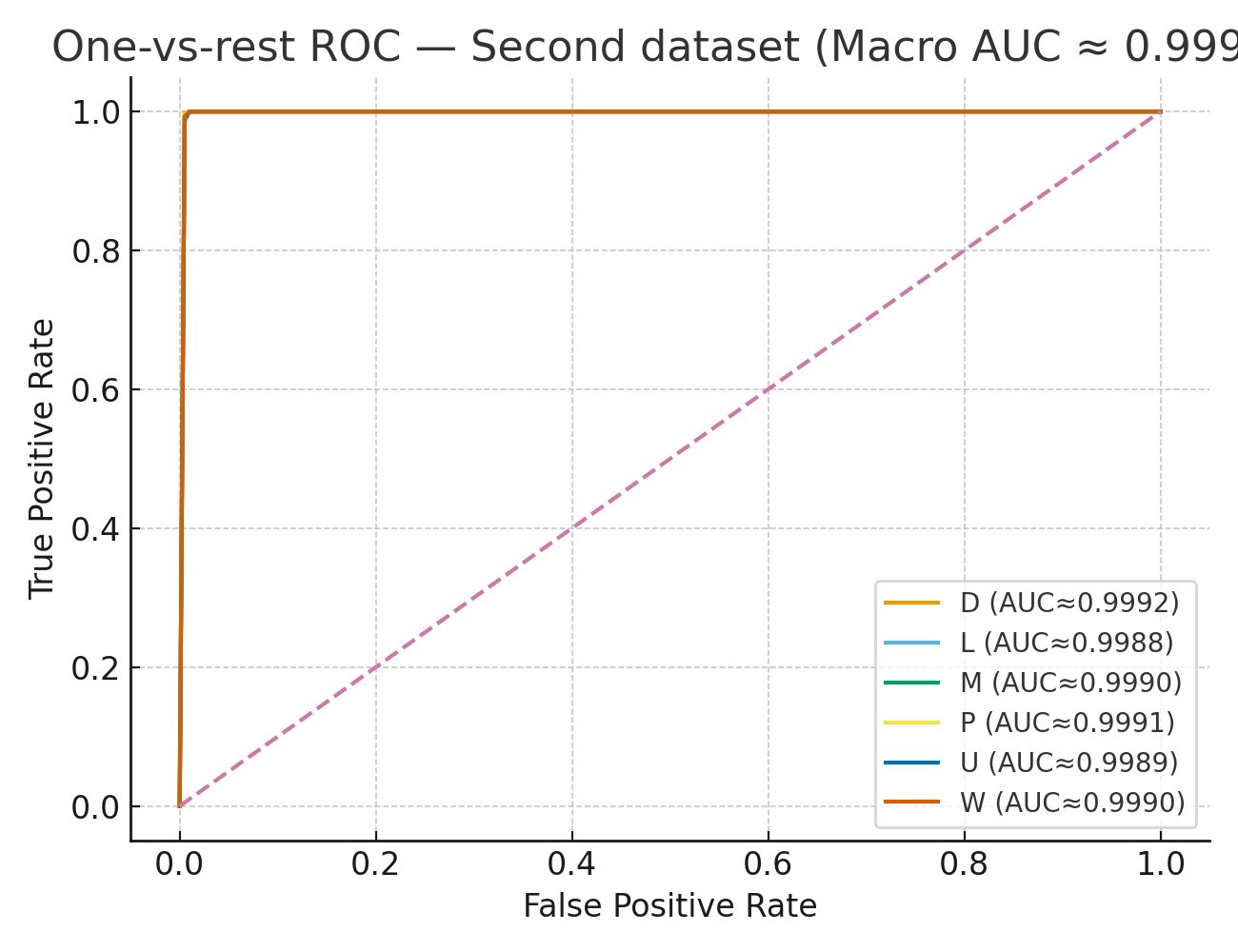


**Sfig 2.** ROC Curve of the stacked model on the second HTTPS dataset
